# *Candida auris*: A systematic review and meta‐analysis of current updates on an emerging multidrug‐resistant pathogen

**DOI:** 10.1002/mbo3.578

**Published:** 2018-01-18

**Authors:** John Osei Sekyere

**Affiliations:** ^1^ Department of Pharmaceutics Faculty of Pharmacy and Pharmaceutical Sciences Kwame Nkrumah University of Science and Technology Kumasi Ghana

**Keywords:** antifungal resistance, C*andida auris*, candidaemia, fungemia, molecular epidemiology

## Abstract

From 2009, *Candida auris* has emerged as a multidrug‐resistant ascomycete yeast pathogen with the capacity for easy transmission between patients and hospitals, as well as persistence on environmental surfaces. Its association with high mortalities, breakthrough and persistent candidaemia, inconsistencies in susceptibility testing results, misidentification by available commercial identification systems and treatment failure, complicates its management and detection. Within the last nine years, *C. auris* has been increasingly reported from far‐Eastern Asia, the Middle East, Africa, Europe, South and North America with substantial fatalities and misidentification. Herein, I provide a systematic and thorough review of this emerging pathogen. Meta‐analysis showed that at least 742 *C. auris* isolates have been reported in 16 countries, with most of these being from India (≥243), USA (≥232) and UK (≥103) (*p*‐value = .0355) within 2013–2017. Most isolates were from males (64.76%) (*p*‐value = .0329) and blood (67.48%) (*p*‐value < .0001), with substantial crude mortality (29.75%) (*p*‐value = .0488). Affected patients presented with other comorbidities: diabetes (≥52), sepsis (≥48), lung diseases (≥39), kidney diseases (≥32) etc. (*p*‐value < .0001). Resistance to fluconazole (44.29%), amphotericin B (15.46%), voriconazole (12.67%), caspofungin (3.48%) etc. were common (*p*‐value = .0059). Commonly used diagnostic tools included PCR (30.38%), Bruker MALDI‐TOF MS (14.00%), Vitek 2 YST ID (11.93%), AFLP (11.55%) and WGS (10.04%) (*p*‐value = .002). Multidrug resistance, high attributable mortality and persistence are associated with *C. auris* infections. Two novel drugs, SCY‐078 and VT‐1598, are currently in the pipeline. Contact precautions, strict infection control, periodic surveillance and cleaning with chlorine‐based detergents, efficient, faster and cheaper detection tools are necessary for prevention, containment and early diagnosis of *C. auris* infections.

## 1 INTRODUCTION

Antimicrobial resistance (AMR) is inarguably one of the greatest threats and challenges to clinical medicine and public health in this century (Laxminarayan et al., 2016). Antimicrobial‐resistant microbes, particularly bacteria and fungi, are increasingly being reported in healthcare and community settings, with high attendant morbidities, mortalities, and healthcare‐associated costs that runs into millions of dollars (Laxminarayan et al., 2016; Osei Sekyere, 2016; Osei Sekyere, Govinden, Bester, & Essack, 2016). Until recently, AMR was mainly reported in bacteria. Specifically, in medically important Gram‐negative ones in which plasmid‐mediated or horizontally acquired antibiotic resistance genes were associated (Nordmann, Jayol, & Poirel, 2016; Osei Sekyere et al., 2016). Notorious genes encoding antibiotic resistance enzymes including extended‐spectrum β‐lactamases (ESBLs) such as CTX‐M, SHV, TEM, GES, and OXA, carbapenemases such as NDM, KPC, IMP, VIM, and OXA‐48 type, and the MCR colistin resistance gene have been raising alerts due to their activity against clinically important antimicrobials (Nordmann, 2014; Osei Sekyere, 2016; Osei Sekyere & Amoako, 2017).

While clinicians are still battling with the above‐stated resistance enzymes in Gram‐negative bacteria, a new multidrug‐resistant ascomycete yeast pathogen emerged in a female patient in Tokyo, Japan, in 2009 and contemporaneously in 15 South Korean patients in the same year (Kim et al., 2009; Satoh et al., 2009). This yeast belonged to the *Candida* genus. As it was detected in the external ear canal of the patient, it was named as *Candida auris*; auris is the Latin word for ear (Satoh et al., 2009). Satoh et al. (2009), who first described this pathogen, found that it clustered in the *Metschnikowiaceae* clade. Further, it was closely related to *Candida lusitaniae, Candida pseudohaemulonii, Candida duobushaemulonii* and *Candida haemulonii. Candida haemulonii* was first isolated from the gut of a blue‐striped grunt fish (*Haemulon scirus*), and later from the blood of a renal failure patient (Cendejas‐Bueno et al., 2012). The closer phylogenetic relationship between *C. auris* and *Candida krusei, C. lusitaniae, C. haemulonii, C. pseudohaemulonii,* and *C. duobushaemulonii,* which are inherently multidrug resistant to amphotericin B (polyenes) and azoles, has been cited as a reason for the similarly higher resistance of *C. auris* to these two drug classes (Cendejas‐Bueno et al., 2012; Lepak, Zhao, Berkow, Lockhart, & Andes, 2017).

Although *C. auris* was initially isolated from the external ear canal or discharges of patients with otitis media, latter reports have shown their involvement in candidaemia/fungemia and other deep‐seated invasive infections with very high associated mortalities and co‐morbidities (Azar, Turbett, Fishman, & Pierce, 2017; Ben‐Ami et al., 2017). Unlike other yeasts, they can be transmitted within and between hospitals, patients and the environment. Furthermore, their resistance to at least one antifungal drug such as the azoles (particularly fluconazole and/or voriconazole), polyenes (amphotericin B), flucytosine, and the echinocandins (caspofungin, micafungin and anidulafungin) is well documented (European Centre for Disease Prevention and Control, 2016; Rudramurthy et al., 2017; Schelenz et al., 2016; Tsay et al., 2017). Various studies have established their persistence in clinical environments, including the air and bedding materials, and even in patients undergoing antifungal treatment (Schelenz et al., 2016; Vallabhaneni et al., 2016). As well, their virulence and pathogenicity have been investigated and found to be almost equal to or a little lesser than that of *Candida albicans* (Ben‐Ami et al., 2017; Borman, Szekely, & Johnson, 2016; Larkin et al., 2017; Sherry et al., 2017); notably, Sherry et al. (2017) found aggregative *C. auris* to be more virulent than *C. albicans* in *Galleria mellonella* larvae (Sherry et al., 2017). Currently, *C. auris* has been reported in 16 countries on five continents: North America (Canada and USA), South America (Colombia and Venezuela), Europe (Germany, Norway, Spain, UK), Africa (South Africa), Asia (India, Israel, Japan, Kuwait, Oman, Pakistan, South Korea) (Chowdhary, Sharma, & Meis, 2017).

Early detection of *C. auris* infections has been shown to be beneficial as earlier initiation of appropriate antifungal therapy saved many lives (Chowdhary et al., 2014; Todd, 2017). However, the inability of several available commercial identification systems/platforms to quickly diagnose *C. auris* remains a challenge to early therapy (European Centre for Disease Prevention and Control, 2016; Kordalewska et al., 2017). While the MALDI‐TOF MS and PCR are currently aiding in this regard with their faster turnaround times, the cost and skill involved in their procurement and operation, respectively, is still a hurdle for most under‐resourced mycology laboratories (Kathuria et al., 2015; Kordalewska et al., 2017; Prakash et al., 2016). There are currently no official therapeutic guidelines, dosage or Clinical Laboratory Standards Institute (CLSI)/European Committee on Antimicrobial Susceptibility Testing (EUCAST) minimum inhibitory concentration (MIC) breakpoints for *C. auris* infections, and studies evaluating these are few (Arendrup, Prakash, Meletiadis, Sharma, & Chowdhary, 2017; Lepak et al., 2017). The sensitivities and specificities of all the diagnostic tools, kits, and media used for detecting this new pathogen are discussed herein.

Microscopic and molecular/genomic analysis have established the presence of phenotypic and genetic/genomic differences between different *C. auris* strains from the same or different regions (Lockhart et al., 2017; Tsay et al., 2017). These include the ability to exist as aggregates or nonaggregate cells, biofilm formation ability, clonality of outbreak strains, and genetic variations between strains from different geographical locations (Borman et al., 2016; Sherry et al., 2017). The virulence characteristics of aggregating and nonaggregating cellular morphologies have been investigated by at least two studies (Borman et al., 2016; Sherry et al., 2017). However, there is much to be done to answer several pending questions about this pathogen and these loopholes are highlighted below. There are currently two novel antifungal drugs that have 100% efficacy against *C. auris:* SCY‐078 from Scynexis pharmaceuticals (Berkow, Angulo, & Lockhart, 2017; Larkin et al., 2017) and VT‐1598 from Viamet pharmaceuticals (Anonymous, 2017).

### 1.1 Purpose of this systematic review

Although there are at least eight excellent reviews addressing this new menace (Table S1), this current work aims to provide a more comprehensive update of *C. auris* reports available to date, and touches on all aspects of the pathogen: phenotypic characteristics, genomic characteristics, virulence and pathogenicity, resistance profiles and mechanisms, crude mortality rates, detection tools and their relative efficiencies, molecular epidemiology, infection prevention and control protocols, and management. It is thus hoped that this work shall become the benchmark reference for all reported findings on *C. auris*.

### 1.2 Databases and keywords used for literature search

**Figure 1:**
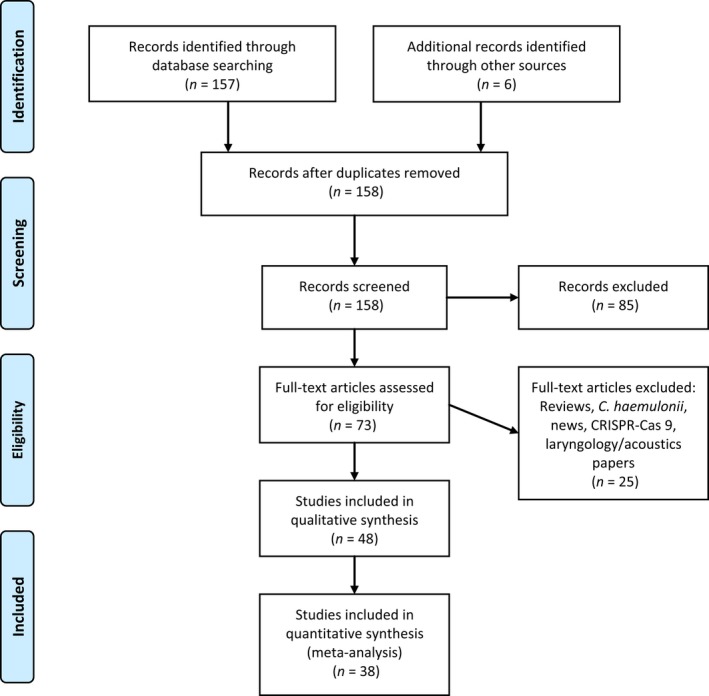
PRISMA‐adapted flow diagram of included and excluded studies. Adapted from the PRISMA website (http://prisma-statement.org/PRISMAStatement/CitingAndUsingPRISMA.aspx) and article

The PRISMA guidelines and checklists (Figure S1) were used in undertaking this systematic review and meta‐analysis. Pubmed, Web of Science and ScienceDirect were searched for English research papers written on *Candida auris* using the search word, *Candida auris*, with the year filter turned to 2009‐01‐01. This returned 157 published articles as at 21/07/2017. Google search, references in returned articles and recently published manuscripts (online) not yet indexed in pubmed were also added to make up to 163 papers. The abstract was screened to remove review articles, non‐English articles, and non‐*Candida auris* papers (Figure 1). Reports on *C. auris* detection or prevalence from the Centers for Disease Control and Prevention (CDC, Atlanta Georgia, USA), Public Health England (PHE) and the European Centres for Disease Control and Prevention (ECDC Stockholm, Sweden) were added. The articles were further categorized into eight as shown in Table S1. All search was done in triplicate to ensure reproducibility.

### 1.3 Statistical analysis

**Figure 2:**
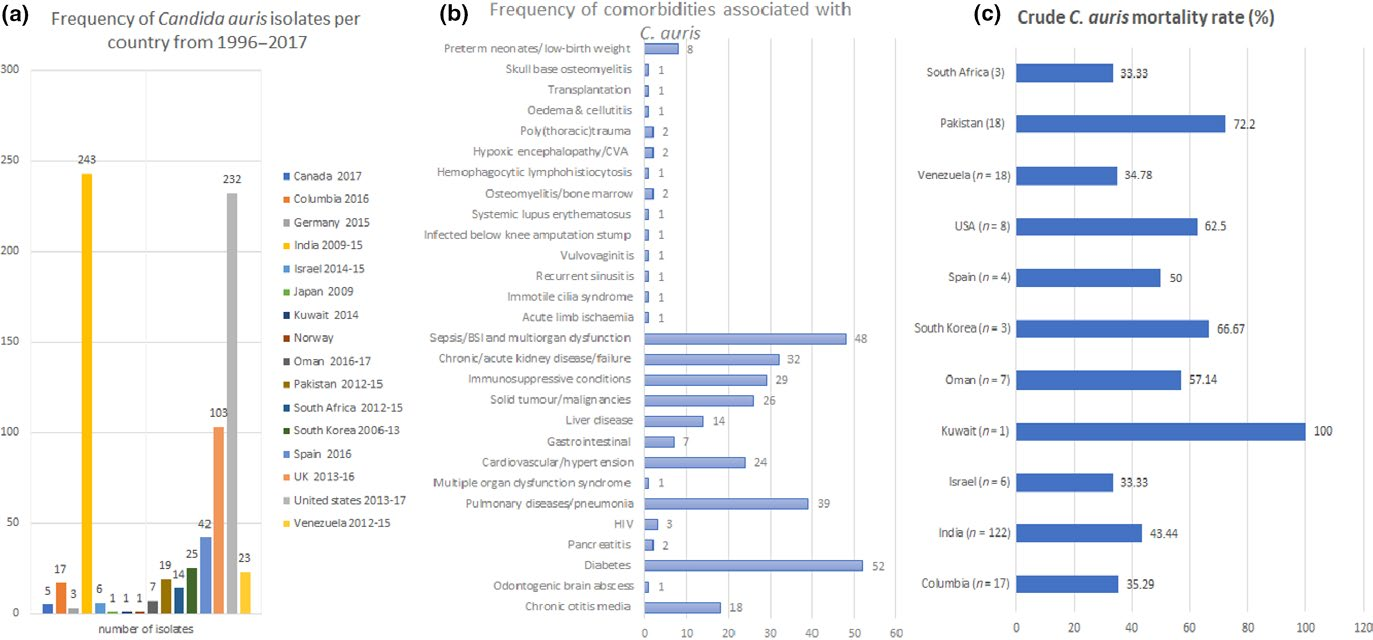
Frequency of *Candida auris* isolated per country between 1996 and 2007 (a), comorbidities presented by *C. auris‐*infected patients (b) and crude mortality rates per country (c). Total number of reported isolates, comorbities, and mortalities per study were collated per country and used to calculate the frequencies. GraphPad was used to calculate the *p*‐values

Unless otherwise stated, tentative MIC breakpoints proposed by the CDC (Centers for Disease Control and Prevention, 2017b) were used for interpretation of the MICs in the meta‐analysis: Resistance to fluconazole (FLZ) ≥32L, amphotericin B (AMB) ≥2, anidulafungin (ANF) ≥4, caspofungin (CFG) ≥2 and micafungin (MCF) ≥4. MICs of all azoles, except FLZ, above 1 mg/L were defined as nonsusceptible (i.e., high and potentially resistant) (Arendrup et al., 2017) and included in the statistics (Figure 2c). Studies that were not specific with the MICs of the individual isolates were excluded from the computation of the resistance rate.

**Figure 3:**
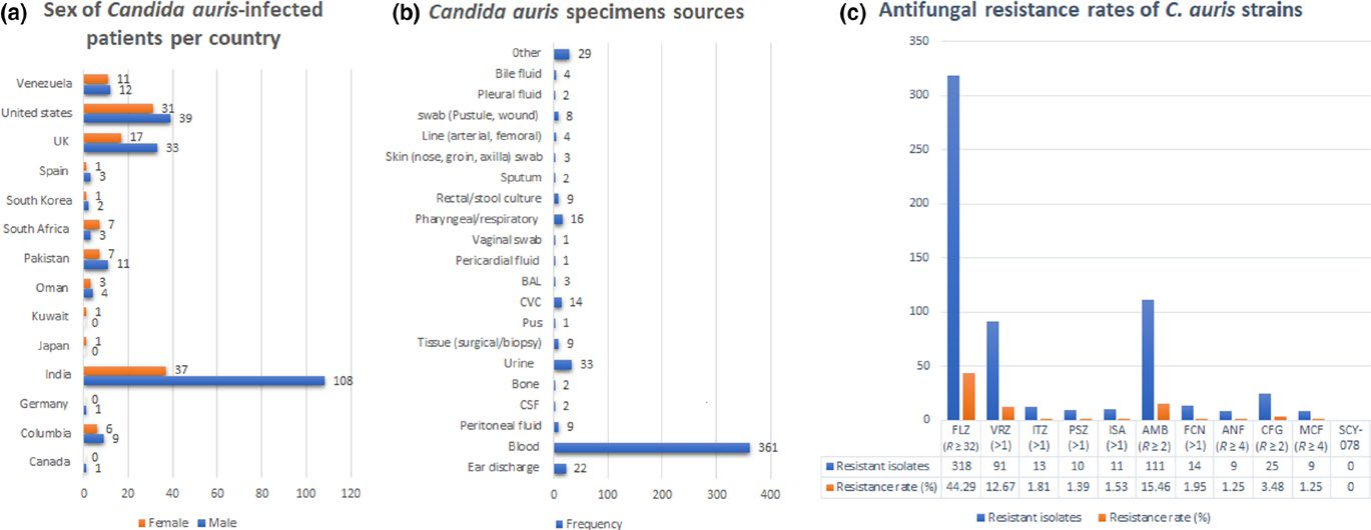
Frequency of males and females infected with *Candida auris* per country (a), specimen sources (b), and antifungal resistance rates (c). Total number of reported cases per male and female patients, specimen sources and antifungal resistance per study were collated per country and used to calculate the frequencies. GraphPad was used to calculate the *p*‐values

The following data were extracted from the included articles: country of detection, year of detection, specimen types obtained from, resistance profiles, diagnostic method used, comorbidities and clinical outcome. These data were imputed into Microsoft Excel and used for the collation of frequencies and charts (Figures 2 and 3). Statistical analysis of the data was undertaken with GraphPad Prism^®^ 5 for Windows, version 5.01 (August 7, 2007). The statistical significance of the data was computed using the Wilcoxon signed rank test and/or the student's *t* test (column statistics or one sample *t* test). The *p*‐values were two‐tailed and calculated with a Gaussian approximation. A *p*‐value of <.05 was defined as significant. Studies that did not provide the required data in the text were excluded from the statistical analysis. All statistical analyses were done in triplicates to ensure reproducibility.

### 1.4 Included articles

The literature search yielded 163 published articles in addition to reports from the CDC, PHE, and the ECDC. Further screening and exclusion reduced these to 48 articles that were used for the write‐up; 38 articles were used for the statistical analysis (Figure 1).

## 2 PHENOTYPIC FEATURES

**Figure 4:**
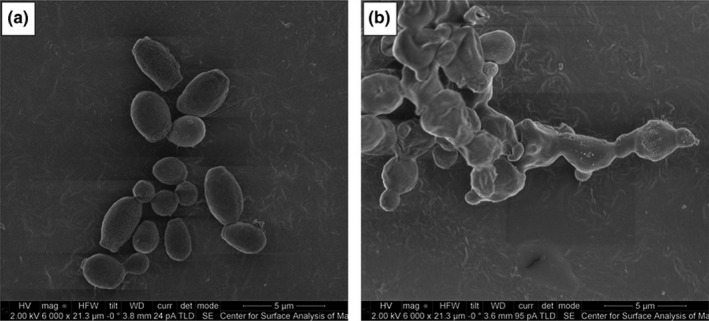
Scanning electron micrograph of *Candida auris* treated with no drug (control) (a) and with SCY‐078 at 1 × MIC (0.5 mg/L) (b). Adapted with permission from Emily Larkin et al. Antimicrob. Agents Chemother. 2017; 61:e02396–16

Microscopy has been instrumental in providing pictorial images of the shapes, color, size, and population structure (Figure 4) of *C. auris* strains growing on different culture media such as Sabouraud's dextrose agar (SDA), CHROMagar, Brilliance *Candida* agar, GYPA culture plates, CS4 agar medium and cornmeal agar at different temperatures and incubation times (Table 1). Particularly on CHROMagar, which is the most common media used, *C. auris* appear as pale purple or pink smooth colonies occurring as single, paired and/or grouped ovoid, ellipsoidal to elongate budding cells (Kathuria et al., 2015; Mohsin et al., 2017; Satoh et al., 2009); on SDA, they appear as smooth white to cream‐colored colonies (Prakash et al., 2016). However, Kumar, Banerjee, Pratap, and Tilak (2015) (Kumar et al., 2015) saw no characteristic color on CHROMagar with their *C. auris* strains, which could be due to the conditions used. The size [(2.0–3.0) × (2.5 × 5.0) μm] and growth rate of *C. auris* is comparable to *Candida glabrata* than to *C. albicans* (Borman et al., 2016), although its growth patterns are similar to *C. albicans* (Larkin et al., 2017). The thermoresistance of *C. auris* that allows it to grow between 30 and 42°C, albeit slowly and weakly at 42°C (Satoh et al., 2009), is a unique characteristic that is unseen in other species of *Candida*. This characteristic can be used in the easy identification of this pathogen from other species and has been cited as a possible reason for the high survival of this pathogen in humans and its potential to survive in avian species (Borman et al., 2016; Chatterjee et al., 2015; Chowdhary et al., 2014; Satoh et al., 2009). Evidently, this thermoresistance will also enhance persistence in the host, aiding in the dissemination of this pathogen in the environment (Piedrahita et al., 2017; Schelenz et al., 2016; Welsh et al., 2017).

**Table 1:** Phenotypic and genomic characteristics of *Candida auris*

Phenotypic and genomic features	Observations	References
Fermented sugars	Glucose, sucrose (weak) and trehalose (weak)	Cendejas‐Bueno et al. (2012), Chowdhary et al. (2013, 2014), Emara et al. (2015), Lee et al. (2011), Satoh et al. (2009)
Nonfermented sugars	Galactose, maltose, lactose or raffinose	
Assimilated carbon sources	Glucose, sucrose, maltose, D‐trehalose, D‐raffinose, D‐melezitose, inulin (weak), soluble starch, ribitol (weak), galactitol, D‐mannitol, sorbitol and citrate, N‐acetyl‐ D‐glucosamine (NAG)^a^	
Nonassimilated carbon sources	D‐galactose, L‐sorbose, D‐cellobiose, lactose, melibiose, D‐xylose, L‐arabinose, D‐ arabinose, ribose, L‐rhamnose, D‐glucosamine, NAG, methanol, ethanol, glycerol, erythritol, α‐methyl‐D‐glucoside, salicin, D‐gluconate, DL‐lactate, succinate, inositol, hexadecane, 2‐keto‐D‐gluconate and xylitol	
Nitrogen sources utilized	Ammonium sulfate, cadaverine, and L‐lysine	
Nitrogen sources not utilized	Sodium nitrite, potassium nitrate and ethylamine are not utilized	
Growth in vitamin‐free medium, 50% glucose, and 10% NaCl/5% glucose medium	Positive	
Growth temperature	37–40°C (optimal); 42°C (weak and slow); >42°C (no growth)	
Starch formation, urease activity and diazonium blue B reaction	Negative	
Growth in the presence of 0.1% and 0.01% cycloheximide	Negative	Cendejas‐Bueno et al. (2012), Chowdhary et al. (2014), Emara et al. (2015), Lee et al. (2011), Rudramurthy et al. (2017), Sarma and Upadhyay (2017), Satoh et al. (2009)
Virulence factors: Hyphae, pseudohyphae, germ tube, and biofilm formation; proteinases and phospholipases^b^ production; adherence	Hyphae formation is negative. Some strains form pseudohyphae occasionally, but most strains do not. No germ tube formed on cornmeal agar. Little adherence to catheter material (compared to *Candida albicans*). Phospholipases (P_*z*_) and proteinases production were strain‐dependent, at different degrees (0.78–1 and 0.0–5.3, respectively) and relatively lower than *C. albicans* (P_z_ = 0.66)	Azar et al. (2017), Borman et al. (2016), Cendejas‐Bueno et al. (2012), Chowdhary et al. (2013, 2014), Kumar et al. (2015, 2017), Larkin et al. (2017), Lee et al. (2011), Satoh et al. (2009), Sherry et al. (2017)
	Most strains form biofilms to different degrees while some do not form biofilms at all^c^	Chatterjee et al. (2015), Chowdhary et al. (2013), Larkin et al. (2017), Oh et al. (2011), Sherry et al. (2017)
Shape, size, appearance chlamydospore and chlamydoconidia formation	Cells are ovoid, ellipsoidal to elongate, (2.0–3.0) × (2.5–5.0) μm, single, in pairs, or in groups/aggregates. Smooth, pale purple, pinkish and creamy colonies on CHROMagar. Some studies saw no characteristic color on CHROMagar. Beige colored colonies formed on Brilliance *Candida* Agar. Obverse colonies white cream on GYPA and Reverse colony milky brown 48 h at 24°C. Obverse colonies nile blue and Reverse light green at 24°C. No chlamydospores or chlamydoconidia were formed on cornmeal agar	Ben‐Ami et al. (2017), Borman et al. (2016), European Centre for Disease Prevention and Control (2016), Kumar et al. (2015), Larkin et al. (2017), Lee et al. (2011), Ruiz Gaitán et al. (2017), Satoh et al. (2009), Schelenz et al. (2016), Sherry et al. (2017)
Misidentification by commercial systems	Vitek 2 YST: *Candida haemulonii, Candida duobushaemulonii*. API 20C: *Rhodotorula glutinis, Candida sake, Saccharomyces cerevisae*. BD Phoenix: *Candida haemulonii, Candida catenulate*. MicroScan: *Candida famata, Candida guilliermondii, Candida lusitaniae, Candida parapsilosis*. Auxacolor 2: *S. cerevisae*	Centers for Disease Control and Prevention (2017b), Chowdhary et al. (2014), Kathuria et al. (2015), Khillan et al. (2014), Kordalewska et al. (2017), Mizusawa et al. (2017), Ruiz Gaitán et al. (2017)
Genomic features	12.3–12.5 Mb genome, GC content = 44.8%–45.3%, CDS^d^ = 6675, 5.8S rRNA, 184 tRNA, 3262 repetitive elements	Centers for Disease Control and Prevention (2017a), Chatterjee et al. (2015), Lockhart et al. (2017), Schwartz and Hammond (2017), Sharma et al. (2016), Tsay et al. (2017), Vallabhaneni et al. (2016)

a Some strains from India, South Africa, Brazil, etc. are able to assimilate NAG (Prakash et al., 2016).

b P_*z*_ < 0.89 (strong phospholipase activity); P_*z*_ = 0.90 to 0.99 (weak phospholipase activity); P_*z*_ = 1 (no phospholipase activity).

c The lack of biofilm formation may be due to several factors: type of substrate and media used, source of isolates (ear/blood), pretreatment with fetal bovine serum (FBS), biofilm measurement/scale used.

d Coding sequence.

In determining the species of this novel *Candida* pathogen*,* Satoh et al. (2009) determined the sugar fermentation and assimilation characteristics of *C. auris*, which has been confirmed by other authors (Table 1) (Satoh et al., 2009). The differences between sugar fermentation and assimilation, nitrogen sources utilization, and high salt tolerance in *C. auris* and other species of *Candida,* has further been used by Welsh et al. (2017) to formulate a highly sensitive and specific Salt Sabouraud dextrose/dulcitol/mannitol and Salt Yeast Nitrogen Base dulcitol/mannitol broths that can easily isolate *C*. *auris* from clinical and environmental specimens (Welsh et al., 2017). Moreover, the inability of *C. auris* to grow on cycloheximide‐containing medium (0.1%–0.01%) (Table 1) could be a marker for the identification of this pathogenic yeast. Thus, the phenotypic and biochemical characteristics of *C. auris,* as detailed in Table 1, can be used in designing novel media and identification kits to enhance the early and efficient detection of this yeast, particularly as misidentification is a major problem with *C. auris* infection management (European Centre for Disease Prevention and Control, 2016; Khillan, Rathore, Kathuria, & Chowdhary, 2014; Lee et al., 2011).

Furthermore, differences exist between strains from Japan and South Korea on one hand, and those from India, South Africa, and Brazil on the other hand in terms of N‐acetyl glucosamine (NAG) utilization (Table 1). This difference has not been fully investigated to ascertain the underlying genetic and/or phenotypic mechanism. Further research should be undertaken to characterize the genetic basis for these differences to aid in a better typing and description of different *C. auris* strains in future.

The inability of *C. auris* to grow pseudohyphae, germ tube, chlamydoconidia, and chlamydospores on cornmeal agar has been established by several researchers (Table 1). However, Borman et al. (2016) and Sherry et al. (2017), respectively, found the formation of rudimentary and occasional pseudohyphae in *C. auris*, suggesting that pseudohyphae formation might be strain‐specific or condition‐specific (Borman et al., 2016; Sherry et al., 2017); further investigations with a larger number of strains will be necessary to comprehensively characterize these differences between strains, the underlying genetic and epigenetic mechanisms or factors and environmental conditions inducing these differences in pseudohyphae formation. The formation of hyphae, pseudohyphae, and germ tube in species such as *C. albicans,* and *Candida tropicalis,* have been associated with higher virulence characteristics (Ben‐Ami et al., 2017; Borman et al., 2016; Larkin et al., 2017) while germ tube and chlamydoconidia formation are used in identifying different fungal or *Candida* spp (Chowdhary et al., 2014; European Centre for Disease Prevention and Control, 2016; Kumar et al., 2015). Thus, the absence of germ tubes, chlamydoconidia/chlamydospores in strains that grow at 42°C, but are unable to grow on NAG‐containing medium should be indicative of *C. auris*. Furthermore, the higher virulence characteristics of *C. auris* even in the absence of pseudohyphae and germ tube formation remains a mystery yet to be unraveled.

Borman et al. (2016), Ben‐Ami et al. (2017), and Sherry et al. (2017) have reported of the presence of at least two cellular morphologies of *C. auris*: aggregating and nonaggregating cells (Ben‐Ami et al., 2017; Borman et al., 2016; Sherry et al., 2017). Borman et al. (2016) showed that aggregating *C. auris* strains could not be separated by mechanical action using vigorous shaking/vortexing and/or chemical treatment with detergents. Thus, it is argued that the aggregating cells are not due to flocculation or encapsulation of cells in biofilms but rather, to the inability of daughter cells to separate after budding. Through *G. mellonella* infection model studies, it has been established that nonaggregating cells are more virulent and pathogenic than aggregating cells and equally, highly or a little less virulent than *C. albicans* (Borman et al., 2016; Sherry et al., 2017). Moreover, nonaggregating *C. auris* cells formed a greater biofilm mass than aggregating ones and *C. glabrata,* and a lower biofilm mass than *C. albicans* (Sherry et al., 2017). Besides the *G. mellonella* infection model studies (Borman et al., 2016; Sherry et al., 2017), no study has shown a higher pathogenicity for *C. auris* over *C. albicans*. Contrasting findings by Larkin et al. (2017) with murine infection models are described below (Larkin et al., 2017). Furthermore, the finding of a higher virulence/pathogenicity among nonaggregating cells than in aggregating cells has only been established in *G. mellonella* models. Thus, additional studies are necessary to establish the relative pathogenicity of these two cellular morphologies in different infection models.

Summing up, *C. auris* has a complicated phenotypic plasticity in terms of cellular morphology, nitrogen and carbon source assimilation and utilization, virulence and pathogenicity, which can be cellular morphology type‐, strain‐ and/or country of origin‐specific. Nevertheless, their ability to grow at 40–42°C has been confirmed worldwide.

## 3 GENOMIC FEATURES

Of the six articles reporting on the use of whole genome sequencing to characterize the genome of *C. auris* (Chatterjee et al., 2015; Lockhart et al., 2017; Sharma, Kumar, Meis, Pandey, & Chowdhary, 2015; Sharma, Kumar, Pandey, Meis, & Chowdhary, 2016; Tsay et al., 2017; Vallabhaneni et al., 2016), only three gave detailed genome characteristics of the sequenced isolates (Chatterjee et al., 2015; Sharma et al., 2015, 2016), which were all from India. In a detailed description of sequenced *C. auris* genomes, Chatterjee et al. (2015), and Sharma et al. (2015, 2016) showed that the *C. auris* genome diverged from that of *C. albicans* by 99.5% and had a size of 12.3–12.5 Mb with a G+C content of 44.53%–44.8% (Chatterjee et al., 2015; Sharma et al., 2015, 2016). Its genome was closest in homology or average nucleotide identity to that of *C. lusitaniae* (85.9%–86.4%), but it lacked the *MATa* mating locus allele, although it had the other allele, *MAT*α. PCR amplification of the *MAT*α gene allowed for easy identification of *C. auris* from other species of *Candida* and can thus be used for identification of *C. auris,* besides the 26S rDNA D1/D2 domain and 18S rRNA internal transcribed spacer (ITS) region DNA (Chatterjee et al., 2015; Satoh et al., 2009; Sharma et al., 2016). Although the *MAT*α allele was found in *C. auris,* its sexuality, that is, parasexual/asexual or sexual, could not be established (Chatterjee et al., 2015; Pragasam et al., 2016); further research is necessary to reveal its sexual cycle.

Within the *C. auris* genome, orthologs of several *C. albicans* efflux genes belonging particularly to the major facilitator superfamily (MFS) and the ATP‐binding cassette (ABC) transporter families were identified, suggesting that efflux is a potential resistance mechanism mediating multidrug resistance (MDR) against azoles, polyenes, and echinocandins in this pathogen (Chatterjee et al., 2015; Sharma et al., 2016). This was phenotypically confirmed by Ben‐Ami et al. (2017) with a rhodamine‐based efflux assay (Ben‐Ami et al., 2017). Further, the zinc (II) 2 cys 6 transcription factor family, of which four members are key regulators of *MDR1,* an efflux pump gene whose upregulation leads to MDR, was enriched in the *C. auris* genome (Chatterjee et al., 2015).

Orthologous genes of *C. albicans* virulence proteins such as STE‐related proteins, MADS‐box, Ste12p, mannosyl transferases, adhesins, and integrins as well as orthologs of *C. albicans* kinases involved in virulence and antifungal stress response such as Hog1 protein kinase, 2‐component histidine kinase etc., were discovered in the *C. auris* genome. Functional annotation of most *C. auris* genes remain to be undertaken and this will be necessary to comprehend the genetic mechanisms of this pathogens’ MDR and virulence/pathogenicity (Grahl, Demers, Crocker, & Hogan, 2017).

Thus, the *C. auris* genome is still not fully characterized and bears little resemblance to the genomes of other species of *Candida*. Several orthologous efflux and virulence genes are present in the genome, but its actual sexual cycle remains a mystery.

## 4 RESISTANCE PROFILES, RATES AND MECHANISMS

The antifungal resistance profiles of the estimated 742 *C. auris* isolates were used to compute the resistance frequency and rates of the isolates to the various antifungals (Figure 2c), using tentative breakpoints developed by the CDC (Centers for Disease Control and Prevention, 2017b) and suggested by Arendrup et al. (2017) (please see Section 1.3). As seen in Figure 2c, most of the isolates were resistant to FLZ (*n* ≥ 318; 44.29%), followed by AMB (*n* ≥ 111; 15.46%), voriconazole (VRZ) (*n* ≥ 91; 12.67%), CFG (*n* ≥ 25; 3.48%), flucytosine (FCN) (*n* ≥ 14; 1.95%), itraconazole (ITZ) (*n* ≥ 13; 1.81%), isavuconazole (ISA) (*n* ≥ 11; 1.53%), posaconazole (PSZ) (*n* ≥ 10; 1.39%), ANF (*n* ≥ 9; 1.25%), MCF (*n* ≥ 9; 1.25%), SCY‐078 (0; 0%) and VT‐1598 (0; 0%). Resistance to at least two of these drugs were frequently reported in several studies (Table 2).

**Table 2:** Geographical distribution, demographics, specimen sources, resistance profiles, diagnostics and clinical data of *Candida auris* isolates identified between 2006/9 and 2017

Country (*n*)	Year (*n*)	Age(*n*)/sex	Specimen source (*n*)	MIC^a^ (μg/ml)	Resistance mechanisms	Diagnostics used	Co‐morbidity	Clinical outcome (*n*)	References
Canada (5)	2017 (5)	64 (1)/M^b^	Ear discharge (1)	FLZ^c^ = 128, AMB^d^ = 2, MCF^e^ = 0.5	ND^f^	MALDI‐TOF MS, WGS^g^	Chronic otitis media, odontogenic brain abscess	Alive	Schwartz and Hammond (2017)
Colombia (17)	2016 (17)	0–77 (9)/M, NS^h^ (6)/F^i^	Blood (13), peritoneal fluid (1), CSF^j^ (1), bone (1), urine (1)	FLZ = 16–>64, VRZ^k^ < 0.12–2, AMB = 8‐>16, MCF < 0.06–0.25, CFG^l^ < 0.25–0.5	ND	API 20C, VITEK 2 YST ID, Phoenix BD, Microscan(Walkaway and AutoSCAN 4), CHROMagar, MALDI‐TOF MS	Diabetes (3), pancreatitis (2), cancer (2), HIV (1)	Demised (6)	Morales‐Lopez et al. (2017)
Germany (2)	NS	68 (1)/M	Blood (2)	SCY‐078^m^ = 0.5, FLZ > 64, ISA^n^ = 0.031, ITZ^o^ = 0.5, PSZ^p^ = 0.25–0.5, VRZ = 0.125–0.5, AMB = 4, FCN^q^ = 0.5, ANF^r^ = 0.25, CFG = 0.5, MCF = 0.25	ND	API 20C AUX, VITEK 2 YST ID, PCR and sequencing (of ITS1/4^s^	NS	NS	Larkin et al. (2017)
Germany (1)	2015 (1)	NS	Blood (1)	NS	ND	NS	NS	NS	European Centre for Disease Prevention and Control (2016)
India (90)	2010–14 (90)	NS	Blood (78), gangrenous tissue (NS), pleural fluid (NS), peritoneal fluid (NS), urine (NS), sputum (NS)	AMB = 0.125–8, ITZ < 0.03–2, VRZ < 0.03–16 ISA < 0.015–4, PSZ < 0.015–8, FLZ = 4–>64 FCN < 0.125–>64, CFG = 0.125–8, MCF < 0.015–8 ANF < 0.015–8	ND, no mutations in *FKS1/2* genes	MALDI‐TOF MS, AFLP, PCR and sequencing (of ITS1, LSU and *RPB1*)	NS	NS	Kathuria et al. (2015), Prakash et al. (2016)
India (74)	2011–12 (74)	49.7 (Mean age); *M* = 46, F = 28, adults=52	Blood (74)	R (FLZ = 43), R (VRZ = 2), R (ITZ = 3), R (CFG = 7), R (AMB = 10)	ND	VITEK 2 YST ID, PCR and sequencing (of ITS1 and D1/D2)	Pulmonary (30), renal (16), cardiovascular (15), gastrointestinal (7), and liver (5) diseases	41.9%–44.7% crude mortalities (19.6%–27% attributable mortalities)	Chakrabarti et al. (2015), Rudramurthy et al. (2017)
India (19), Pakistan (19), South Africa (10), Venezuela (5),	2012–15 (53)	24–69 (53)/M = 26, F = 15, NS=13	Blood (*n* = 27), urine (*n* = 10), tissue (*n* = 5) or other (*n* = 11)	FLZ = 4–256, VRZ = 0.03–16, ITZ = 0.125–2, PSZ = 0.06–1, CFG = 0.03–16, ANF = 0.125–16, MCF = 0.06–4, FCN = 0.125–128, AMB = 0.38–4	*ERG11* mutations: *F126T*, Y132F, Y132F, K143R	WGS	Diabetes (34), solid tumor (12), liver disease (8), immunocompromised (20)	Demised (24)	Lockhart et al. (2017)
India (17)	2013–14 (17)	NS	NS	NS	ND	VITEK 2 YST ID, VITEK 2 MS, PCR and sequencing (of 18S rRNA)	NS	NS	Wattal et al. (2017)
India (15)	2011–13 (15)	48, 80 &87 (3)/F2 (1)/M, 20–79 (8)/M	Blood (7), pus (1), CVC^t^ (3), surgical tissue (3), Broncho alveolar lavage (BAL) (1)	FLZ = 64, VRZ = 0.5–4, FCN = 0.25–64, CFG = 0.25–1, PSZ = 0.015–0.125, ITZ = 0.06–0.25, ISA = 0.06–0.5, AMB = 0.25–1, MCF = 0.06–0.125, ANF = 0.125–0.25	ND	CHROMagar, PCR and sequencing (of ITS and D1/D2)	Diabetes (5), chronic kidney disease (4), malignancies (3), sepsis (4), acute renal failure (2), chronic kidney disease (3), (broncho‐)pneumonia (2), peripheral occlusive vascular disease (3), IgA nephropathy, hydronephrosis etc.	Demised (4)	Chowdhary et al. (2014)
India (12)	2009–11 (12)	NS	Blood (12)	FLZ = 16–64, AMB = 0.25–1, ITZ = 0.125–0.25, VRZ = 0.125–1 ISA < 0.015–0.25, PSZ = 0.06–0.25, FCN = 0.125, CFG = 0.125–0.25, MCF = 0.06–0.125, ANF = 0.125–0.5	ND	API 20C, VITEK 2 YST ID, PCR and sequencing (of ITS1 and D1/D2), AFLP^u^	Immunosuppressive conditions (7), diabetes (6), CKD^v^, cancer chemotherapy (2), HIV (1), low birth weight (3), sepsis (1), acute lymphoblastic leukemia (1)	Demised (6)	Chowdhary et al. (2013)
India (5)	2012–14 (5)	NS	Blood (5)	FLZ = 16–64, AMB = 4–16, FCN = 1, CFG = 0.25	ND	VITEK 2 YST ID, WGS, PCR (MFα)	Sepsis and multiorgan dysfunction (1)	NS	Chatterjee et al. (2015)
India (4)	2013 (4)	43/M	Pericardial fluid (1), blood (1), BAL (1) and urine (1)	AMB = 0.125–0.5, CFG = 1, FLZ > 64, PSZ ≤ 0.015, ITZ = 0.03–0.125, VRZ = 0.06–0.125, FCN = 0.125–4, MCF = 0.06, ANF = 0.125–0.25	ND	CHROMagar, Vitek 2, PCR and sequencing (of ITS and D1/D2)	Chronic liver disease	Demised (1)	Khillan et al. (2014)
India (3)	2013–14 (3)	NS	Blood (3)	NS	ND	MALDI‐TOF MS, PCR and sequencing (of ITS1 and D1/D2)	NS	NS	Ghosh et al. (2015)
India (2)	2011 (2)	NS	NS	FLZ = 64, VRZ = 2, AMB = 16, FCN = 1	ND	Vitek 2, PCR and sequencing (of ITS and D1/D2)	NS	Demised (≤2)	Sarma et al. (2013)
India (1)	NS	28 (1)/F	Vaginal swab (1)	ITZ ≥ 2FLZ ≤ 16, VRZ ≤ 0.5 and AMB ≤ 0.5	ND	CHROMagar, PCR and sequencing (of ITS1)	Vulvovaginitis	Survived (1)	Kumar et al. (2015)
India (1)	2015	NS	Blood (1)	FLZ = 64	ND	Vitek 2, PCR and sequencing (of ITS and D1/D2, RPB1/2)	NS	NS	Sharma et al. (2015)
Israel, Tel Aviv (6)	2014 (4), 2015 (1), 2014–15 (1)	NS	Blood (5), NS (1)	FLZ = 32–64, ITZ = 0.5, VRZ = 0.5–1, PSZ = 0.12–0.5, AMB = 1–2, ANF = 0.03, MCF = 0.12–0.25, CSF = 0.5, FCN = 0.25–1	ND, higher ABC efflux activity	CHROMagar *Candida*, VITEK 2 YST ID, PCR and sequencing	HIV (1), blood stream infections (5)	Demised (2)	Ben‐Ami et al. (2017)
Japan (1)	2009 (1)	70 (1)/F	Ear discharge (1)	FLZ = 2, VRZ = 0.031, ITZ = 0.063, FCN = 0.5	ND	CHROMagar, Vitek 2, PCR and sequencing (of ITS and D1/D2)	NS	Alive	Satoh et al. (2009)
Kuwait (1)	2014 (1)	27/F	Blood (1)	FLZ ≥ 256, AMB = 0.064, VRZ = 0.38, CFG = 0.0064	ND	VITEK 2, MAST ID CHROMagar, PCR (of ITS1 and D1/D2)	Chronic renal failure, lobar pneumonia, immotile cilia syndrome, bronchiectasis, recurrent sinusitis	Demised	Emara et al. (2015)
Norway (1)	NS (1)	NS	Blood (1)	NS	ND	NS	NS	NS	European Centre for Disease Prevention and Control (2016)
Oman (5)	2016–17 (5)	62 (2)/M, 71 (1)/M, 31 (1)/F, 62 (1)/F	Blood (5)	FLZ = 128‐>256, VRZ = 0.5–2, ITZ = 0.12–0.25, PSZ = 0.06–0.12, ANF = 0.12, CFG = 0.08–0.12, MCF = 0.06–0.12, AMB = 1–2, FCN = 0.06–8	ND	BD Phoenix Yeast ID panel	Diabetes mellitus (2), cerebrovascular accident (1), chronic kidney disease (1), sepsis (1), acute limb ischemia (1), metastatic endometrial cancer (1), obstructive uropathy (1), infected below knee amputation stump (1), kidney transplant (1), systemic lupus erythematosus (1), pneumonitis (1)	Demised (3)	Al‐Siyabi et al. (2017)
Oman (2)	2016 (1), 2017 (1)	70 (1)/F, 77 (1)/M	Blood (2)	FLZ ≥ 64, ITZ = 0.125–0.031, VRZ = 0.125–1, PSZ < 0.016–0.125, ISA < 0.016–0.125, AMB = 1–2, ANF = 0.031–0.125, MCF = 0.063–0.125	ND	API20C‐ AUX, MALDI‐TOF MS, PCR and sequencing (of ITS and LSU rRNA), AFLP	Diabetes, hypertension, cardiac failure, edema and cellutitis (1), diabetes, osteomyelitis and septic shock (1)	Demised (1)	Mohsin et al. (2017)
South Africa (4)	2012–13 (4)	85 (1), 73 (1), 60 (1), 27 (1)	Blood (4)	FLZ = 64‐>256, VRZ = 0.25–2, PSZ = 0.015–0.06, ITZ = 0.06–0.25, FCN = 0.06–0.12, CFG = 0.03–0.25, MCF = 0.06–0.12, ANF = 0.06–0.25	ND	API 20C, VITEK 2 YST ID, PCR and sequencing (of ITS1 and D1/D2)	NS	NS	Magobo et al. (2014)
South Korea (20)	2006 (15), 2007–10 (5)	NS	Ear (17), blood (3)	FLZ = 2–128, AMB = 0.38–1.5, ITZ = 0.125–4, VRZ = 0.03–2, CFG = 0.125–0.25, MCF = 0.03	ND	VITEK 2 YST ID, PCR and sequencing (of ITS1 and D1/D2)	Otitis media (17), candidaemia (3)	Survived (17), NS (3)	Kim et al. (2009), Oh et al. (2011), Shin et al. (2012)
South Korea (3)	1996 (1), 2009 (2)	1 (1)/F, 1 (1)/M, 74 (1)/M	Blood (3)	AMB = 0.5–1, FLZ = 2–128, ITZ = 0.125–2, VRZ = 0.06–1, CFG = 0.06, MCF = 0.03	ND	API 20C, VITEK 2 YST ID, PCR and sequencing (of ITS1 and D1/D2)	Hypoxic encephalopathy and aspiration pneumonia (1), laryngeal carcinoma (1), hemophagocytic lymphohistiocytosis (1)	Demised (2)	Lee et al. (2011)
South Korea (2)	2010–13 (2)	NS	Ear discharge (2)	NS	ND	Phoenix BD system, VITEK 2 YST ID, MALDI TOF MS (VITEK MS and Bruker) PCR and sequencing (of ITS1 and D1/D2)	NS	NS	Kim et al. (2016)
Spain (34)	2016 (34)	NS	Blood (34)	NS	ND	NS	Blood stream infection (34)	NS	European Centre for Disease Prevention and Control (2016)
Spain (8)	2016 (8)	66 (1)/M, 48 (1)/M, 26 (1)/M, 39 (1)/F,	Blood, CVC tip, urine, peritoneal fluid, pharyngeal, and rectal culture (4)	FLZ > 256, VRZ = 2, susceptible to PSZ, ITZ, MCF, ANF and AMB	ND	CHROMagar *Candida* ^®^, BBL Mycosel agar, API ID20C, AuxaColor, VITEK MS, PCR & sequencing of ITS	Hepatocellular carcinoma (1), ventricular dysfunction and multiple organ dysfunction syndrome (1), poly(thoracic)trauma (2),	Alive (2Demised (2)	Ruiz Gaitán et al. (2017)
UK (53)	2013 (3), 2014 (1), 2015 (7), 2016 (4), 2014–16 (7)	NS	Blood (7), sputum (2), groin swab (2), CSF (1), NS (18), line (1), arterial line (1), pleural fluid (2), urine (1), pustule swab (1), wound swab (3), femoral line (2), swab (2)	FLZ = 8−>64, VRZ = 0.06–2, PSZ = <0.03–1, ANF = 0.03–0.5, FCN = <0.125–0.25, AMB = 05–1	ND	PCR (of 28s rRNA/ITS1), MALDI‐TOF MS	NS	NS	Borman et al. (2016, 2017)
UK (50)	2015–16 (50)	19–78 (50)/M = 33, F = 17	Wound swabs, urine samples, vascular devices tips, blood cultures, skin (nose, axilla, groin) stool samples	FLZ > 256, AMB = 0.5–2M, FCN <0.06–0.12, ANF/MCF/CFG = 0.06–0.25	ND	Brilliance *Candida* Agar, MALDI‐TOF, AFLP	Cardiac surgery	Survived (50)	Schelenz et al. (2016)
United states (224: 104 are clinical, 120 are colonized patients)	2016–17 (224)	21–96 (69)/55%M	Blood (40), urine (10), respiratory tract (8), bile fluid (4), wound (1), CVC tip (2), bone (1), jejunal biopsy (1)	R^w^ (FLZ>32) = 30, R(AMB≥2) = 15, R(MCF/ANF/CFG >4) = 1	ND	WGS	NS	NS	Centers for Disease Control and Prevention (2017a), Tsay et al. (2017)
United States (7)	2013 (1), 2015 (1), 2016 (5)	Not specified (NS)	Blood (5), urine (1), external ear canal (1)	R (FLZ) = 5 isolates, R(AMB) = 1, R (MCF/ANF/CFG) = 1	ND	WGS	Hematologic malignancies (*n* = 2), bone marrowtransplantation (*n* = 1),acute respiratory failure (*n* = 1), peripheral vascular disease and skull base osteomyelitis (*n* = 1), brain tumor, villous adenoma resection (1).	Demised (4), alive (3)	Vallabhaneni et al. (2016)
United States, Massachusetts (1)	2017 (1)	71(1)/M	BAL	FLZ = 4, VRZ = 0.03, CFG = 0.12, MCF = 0.12, FCN = 0.12, AMB = 2	ND	CHROMagar *Candida*, VITEK MS, VITEK 2 YST ID, MALDI‐TOF MS	Idiopathic pulmonary fibrosis, chronic obstructive lung disease	Demised	Azar et al. (2017)
Venezuela (18)	2012–13 (18)	<1 year (6)/M<1 year (6)/F14 (1)/F72 (1)/F21–29 (2)/M40–48 (2)/M	Blood (18)	FLZ > 64, VRZ = 4, AMB = 2, FCN = 0.5, ANF = 0.125	ND	VITEK 2 YST ID, PCR and sequencing (of ITS), AFLP	Preterm neonates (8), cancer (1),	Demised (5)	Calvo et al. (2016)

a Minimum inhibitory concentration. Tentative MIC breakpoints proposed by the CDC (Centers for Disease Control and Prevention, 2017b) were used for interpretation: Resistance to FLZ ≥ 32L, AMB ≥ 2, ANF ≥ 4, CFG ≥ 2 and MCF ≥ 4.

b Male.

c Fluconazole.

d Amphotericin B.

e Micafungin.

f Not determined.

g Whole genome sequencing.

h Not stated.

i Female.

j Cerebrospinal fluid.

k Voriconazole.

l Caspofungin.

m A novel orally bioavailable 1,3‐β‐D‐glucan synthesis inhibitor antifungal drug.

n isavuconazole.

o Itraconazole.

p Posaconazole.

q Flucytosine.

r Anidulafungin.

s Internal transcribed spacer region.

t Central venous catheter.

u Amplified fragment length polymorphism.

v Chronic kidney disease.

w Resistance: R (FLZ) = fluconazole resistance, R (AMB) = amphotericin B resistance.

Although susceptible *C. auris* strains, specifically to FLZ, have been described (Vallabhaneni et al., 2016), most *C. auris* strains have been reported to be resistant to FLZ and/or to other azoles such as VRZ and to AMB, with a minority being resistant to FCN, other azoles and the echinocandins (Table 2; Figure 2). In several cases, MDR to FLZ and AMB or to all three antifungal drug classes (azoles, polyenes and echinocandins) have been reported (Table 2) (Chakrabarti et al., 2015; Lockhart et al., 2017). The order of resistance as shown in Figure 2 namely, FLZ > AMB > echinocandins, is the same in most of the studies reported so far in most countries (Arendrup et al., 2017; European Centre for Disease Prevention and Control, 2016; Todd, 2017) (Table 2). Thus, higher resistance to FLZ in a *Candida* nonalbicans species has become one of the distinguishing characteristics indicative of a potential *C. auris* infection (European Centre for Disease Prevention and Control, 2016). Due to the relatively low resistance to echinocandins, it is recommended that an echinocandin empirical therapy be initiated in patients suspected to have *C. auris* infections prior to antifungal susceptibility testing of collected strains (Lee et al., 2011; Todd, 2017). The echinocandins can then be maintained or changed based on the susceptibility results (Lepak et al., 2017; Todd, 2017); it should, however, be noted that some patients have died even while on echinocandins (Azar et al., 2017; Ruiz Gaitán et al., 2017; Schelenz et al., 2016). Early initiation of echinocandin therapy has been advised to cut down on *C. auris‐*mediated mortalities (Chowdhary et al., 2014; Lee et al., 2011).

Resistance to other azoles such as ITZ, ISA and PSZ has been variable (Table 2). Arendrup et al. (2017) suggested that the variable resistance to other azoles besides FLZ might be due to a mixed population of resistant and susceptible (or wild‐type and nonwild‐type) *C. auris* strains or the presence of different resistance mechanisms within the population being tested. In other words, the collective resistance mechanism(s) found in the various strains making up the population can affect the final MIC (Arendrup et al., 2017). Caution should be exercised in interpreting MIC data for AMB and CFG generated by Vitek 2 as substantial discrepancies (higher AMB and lower CFG MICs) has been reported between MICs generated by the CLSI's microbroth dilution (MBD) method and the Vitek 2 instrument (Kathuria et al., 2015; Khillan et al., 2014).

A comprehensive characterization of *C. auris* resistance mechanism(s) is currently unavailable although few researchers have attempted to provide some insights. Oh et al. (2011) earlier reported that *C. auris* form no biofilms, an important AMR mechanism. However, this has been discounted by several authors (Larkin et al., 2017; Sherry et al., 2017) and biofilm‐forming genes have been identified in *C. auris* genomes (Chatterjee et al., 2015). The type of substrate and media used, source of isolates (ear/blood), pretreatment with fetal bovine serum (FBS), biofilm measurement/scale used etc. are reasons suggested to have led to the different observations recorded by Oh et al. (2011) on the nonformation of biofilms by *C. auris* (Larkin et al., 2017). Sherry et al. (2017) showed that *C. auris* biofilms, just like that of other species of *Candida,* were resistant to CFG and MCF (MIC > 32 mg/L), to FLZ (MIC > 32 mg/L), to VRZ, and AMB (MIC > 4 mg/L); only liposomal AMB was effective in limiting growth at a lower concentration (MIC = 0.25–1 mg/L), albeit up to 16 mg/L was necessary to stop biofilm metabolic activity by 90% (Sherry et al., 2017).

The direct role of efflux pumps in *C. auris* antifungal resistance is yet to be comprehensively characterized although Ben‐Ami et al. (2017) used rhodamine, an efflux substrate, to show that *C. auris* expressed a higher ABC‐type efflux pump activity than *C. glabrata* and *C. haemulonii* (Ben‐Ami et al., 2017). This higher efflux activity suggested that efflux pumps play an important role in *C. auris* MDR mechanisms, which is corroborated by the several MFS and ABC‐type efflux pumps’ orthologous genes identified by Chatterjee et al. (2015) in *C. auris* genomes (Chatterjee et al., 2015).

Furthermore, the role of mutations in *ERG3* and *ERG11* genes in conferring resistance to azoles in *C. auris* has been investigated (Chatterjee et al., 2015; Lockhart et al., 2017; Sharma et al., 2016) by aligning orthologs of these genes in *C. auris* to that of susceptible *C. auris* and/or *C. albicans* and calling SNPs. The presence of known resistance‐conferring mutations and/or hotspots in *C. albicans’ ERG11* in *C. auris* orthologs have been inferred as a possible resistance mechanism (Lockhart et al., 2017). However, transcomplementation or functional studies of these mutated genes have not been undertaken to establish the effect of these mutations in *C. auris,* specifically in terms of MIC effects. In addition, no known resistance‐conferring mutations in the *FKS* gene have been identified to date and the *ERG11* mutations identified by Lockhart et al. (2017), that is, F126T in South African strains, Y132F in Venezuelan strains, and Y132F or K143R in Indian and Pakistani strains, were found to be clonally and geographically related (Lockhart et al., 2017). A comprehensive study on the resistance mechanisms of *C. auris* is required to decipher the MDR nature of this pathogen.

Hence, it is obvious that efflux, mutations in the *ERG* and *FKS,* and biofilm formation are potential *C. auris* resistance mechanisms. In addition, *C. auris* is generally resistant to FLZ, moderately resistant to AMB, and variably resistant to other azoles, flucytosine and echinocandins.

## 5 VIRULENCE AND PERSISTENCE

Kumar et al. (2015) first undertook phospholipase, proteinase, and hemolysin activity assays in *C. auris* to evaluate their virulence in vitro. Phospholipases, proteinases, and hemolysins are important enzymes that are used by fungi to invade and infect the host (Kumar et al., 2015; Larkin et al., 2017). In that report, substantial phospholipase activity (P_z_ value = 0.72), proteinase activity (P_rz_ value = 0.66) and hemolysin activity (H_z_ value = 0.74) were recorded in the single *C. auris* isolate; a P_z_, P_rz_ or H_z_ value of 1 represents no activity (Kumar et al., 2015; Larkin et al., 2017). The presence of several virulence genes in *C. auris* genomes has also been attested to (Chatterjee et al., 2015; Sharma et al., 2016). As already noted, no germ tubes were formed by the isolate on corn meal agar. These findings were recently followed up by Larkin et al. (2017) with a larger number (*n* = 16) of isolates in which they observed that not all *C. auris* strains expressed phospholipases and proteinases, and none produced germ tubes (germinated) upon incubation with fetal bovine serum. Moreover, even among strains that did express the virulence proteins, the degree of activity was not the same but strain‐specific, showing that not all *C. auris* strains are virulent/pathogenic or equally virulent/pathogenic. As well, two representative strains showed relatively poorer adherence to catheters than *C. albicans,* suggesting that adherence to catheters could not be a major means/cause of invasive *C. auris* infections and persistence in patients and hospitals (Larkin et al., 2017). However, there are reports on the clearance of *C. auris* candidaemia upon removal of urinary or central venous catheters from patients (Chowdhary et al., 2014; Lee et al., 2011; Ruiz Gaitán et al., 2017).

It is currently agreed that *C. auris* forms relatively less biofilms in terms of biomass and metabolic activity than *C. albicans*, with nonaggregating *C. auris* strains forming more biofilm mass than aggregating ones (Larkin et al., 2017; Sherry et al., 2017). Whereas *C. auris* biofilms have been shown to be resistant to FLZ, VRZ, echinocandins, and AMB (Sherry et al., 2017), the biofilms were found to be composed of very limited extracellular matrix, relatively thin and composed mainly of yeast cells (Larkin et al., 2017). Notably, orthologous biofilm‐forming genes of *C. albicans* such as aspartyl proteases genes, the essential phosphatidyl inositol kinase gene (PIK), the essential poly (A) polymerase gene (PAP), and the nonessential oxysterol‐binding protein gene (OBP) have been found in *C. auris* genomes (Chatterjee et al., 2015; Sharma et al., 2016).

Borman et al. (2016) first reported of two different *C. auris* cellular morphologies based on cell aggregation and showed that nonaggregating cells are equally or a little less virulent than *C. albicans*, the model pathogenic species of this genus (Borman et al., 2016). This was seconded by Sherry et al. (2017) that nonaggregating cells can be more virulent and pathogenic than *C. albicans* (Sherry et al., 2017). Further, Borman et al. (2016) showed that hyphae and pseudohyphae formation are important virulent factors in *Candida* in that nonhyphae and nonpseudohyphae‐forming species such as *C. glabrata, Candida kefyr, C. krusei,* and *Saccharomyces cerevisiae* were less virulent and pathogenic than hyphae‐forming ones such as *C. albicans* and *C. tropicalis* and the rudimentary pseudohyphae‐forming pathogen, *C. auris* (Borman et al., 2016). This was evident from the survival times recorded in *G. mellonella* infection models. Larkin et al. (2017) however, contend that the use of murine infection models indicates that *C. auris* is far less virulent than *C. albicans* and that the MDR nature of *C. auris* is a fitness cost for its reduced virulence compared to *C. albicans*. Moreover, they asserted that *C. auris* could not effectively infect and disseminate in mice unless they were immunocompromised, and a larger *C. auris* inoculum size (3 × 10^7^ yeast cells/animal) was administered (Larkin et al., 2017). In contrast, a higher virulence and pathogenicity of *C. auris* in mice was suggested by the findings of Ben‐Ami et al. (2017), but with aggregating cells (Ben‐Ami et al., 2017). The possibility that different infection models might yield different virulence results should be considered in future virulence and infection model studies.

One of the alarming characteristics of *C. auris* is its ability to persist on both dry and moist surfaces, bedding materials, floors, sinks, the air, beds, on the skin, in nasal cavities and internal tissues of patients etc. (Piedrahita et al., 2017; Schelenz et al., 2016; Vallabhaneni et al., 2016; Welsh et al., 2017). Piedrahita et al. (2017) showed the ability of *C. auris* to colonize and spread from hospital environments by growing them on moist and dry surfaces for at least 7 days. Moist surfaces produced more *C. auris* colonies than dry ones and their recovery from dried surfaces was similar to that of other species of *Candida,* methicillin‐resistant *Staphylococcus aureus* (MRSA), vancomycin‐resistant *Enterococcus* (VRE) and carbapenem‐resistant *Enterobacteriaceae* (CRE) (Piedrahita et al., 2017). However, *C. auris* was recovered at a higher rate than *C. albicans,* but significantly less than *Candida parapsilosis* (Piedrahita et al., 2017). Further, Welsh et al. (2017) also evaluated the persistence of *C. auris* vis‐à‐vis *C. parapsilosis* on plastic surfaces and found that *C. auris* can persist for at least 2 weeks on culture and 1 month when their esterase activity (viability) is measured with a solid‐phase cytometer (Welsh et al., 2017).

The higher sensitivity of the esterase activity test, which can identify single cells, makes it ideal for testing the sterility of sterile products and determining the presence of *C. auris* in hospital environments; it should thus be used alongside culture‐based surveillance. This is because *C. auris* failed to grow on culture after 2 weeks on plastic surfaces while the esterase activity test continually remained positive for an additional 2 weeks. Furthermore, while the cultured *C. auris* isolates from plastic surfaces grew for 2 weeks, *C. parapsilosis* grew for 1 month; however, the esterase activity test showed that *C. auris* persisted for a least a month and was more viable than *C. parapsilosis* (Welsh et al., 2017). Persistence times between resistant and susceptible *C. auris* strains need further investigation. And the potential of culture‐negative but esterase activity‐positive (viable) strains to cause infection and hospital spread should be interrogated.

*Candida auris* can colonize, persist and recur in patients several months after first detection, allowing it to be distributed or spread to other patients and in hospitals; even more worrying is the persistent presence of a susceptible *C. auris* strain in the urine of a patient on FLZ treatment (Vallabhaneni et al., 2016). It is estimated that ≥4 hr is the minimum contact period for acquisition of *C. auris* from an infected person or surface (Schelenz et al., 2016). Moreover, *C. auris* can colonize and be shed from the skin at a rate of approximately 10^6^ cells/hr, leading to prolonged outbreaks and transmissions in hospitals (Schelenz et al., 2016; Welsh et al., 2017). It is thus not surprising that *C. auris* has been found on bedding materials, catheter tips and other medical devices, in the air, on window sills, floors, on neighboring patients, etc. in infected patients’ wards (European Centre for Disease Prevention and Control, 2016; Schelenz et al., 2016; Tsay et al., 2017; Welsh et al., 2017). During a hospital outbreak in the UK, for instance, a nurse who was caring for a heavily infected patient was found to be colonized with the same *C. auris* strain as that of the patient in the nose, but this was cleared after receiving oral nystatin, nasal ointment and continual chlorhexidine washes; the nurse obtained the *C. auris* colonization from the patient. Fortunately, the nurse was only transiently colonized and did not transfer the strain to other patients or staff (Schelenz et al., 2016). Even among patients on echinocandins therapy, candidaemia and skin colonization occurred, showing the difficulty in clearing *C. auris* infections (Schelenz et al., 2016). *Candida auris* has been isolated from the axilla and groins of patients and swabbing of these regions are recommended for *C. auris* surveillance (Vallabhaneni et al., 2016; Welsh et al., 2017). In all, these show the ability of *C. auris* to inhabit and persist in various niches, and corroborates the need to periodically surveil and disinfect healthcare settings previously infected with *C. auris*.

In conclusion, *C. auris* persists in a viable form on dried or moist surfaces for several weeks longer than *C. albicans* and *C. parapsilosis*. It forms lesser biofilm mass than *C. albicans,* has poorer adherence to catheters, produces no germ tubes and has strain‐specific expression of hemolysins, proteinases and phospholipases virulence factors.

## 6 DEMOGRAPHICS (SEX, AGE), RISK FACTORS (COMORBIDITIES), MORTALITY RATES AND SPECIMEN SOURCES

An estimated 742 *C. auris* isolates from at least 340 patients were calculated from all the published articles (*n* = 38) and reports of CDC, PHE and ECDC up to the time of writing this article (11–27/08/2017). The five continents and 16 countries with reported *C. auris* cases consisted of North America (Canada and USA), South America (Colombia and Venezuela), Europe (Germany, Norway, Spain, UK), Africa (South Africa), and Asia (India, Israel, Japan, Kuwait, Oman, Pakistan, South Korea) (Figure 2). India (*n* ≥ 243), the United States (Centers for Disease Control and Prevention, 2017a; Tsay et al., 2017) (*n* ≥ 232) and the United Kingdom (*n* ≥ 103) reported the highest number of isolates and infected and/or colonized patients to date (Figure 2; Table 2) (*p*‐value = .0355).

The reported *C. auris* isolates were mostly isolated from males (*n* ≥ 226, 64.76%) while 35.24% (*n* ≥ 123) were from females (Figure 3) (*p*‐value = .0329). In all countries except South Africa, there were more male *C. auris‐*infected patients than females. Further, the differences between male and female *C. auris‐*infected patients were marginal (<10 patients difference) in all countries except the UK (difference of 16 patients) and India (difference of 71 patients). No reason has been provided yet for the sexual differences in terms of frequency of *C. auris* infections. However, *C. auris* case differences between sexes are country‐specific and local health factors might play a role in the higher male rates recorded per country and worldwide. In addition, most of the reported cases of *C. auris* occurred or escalated within the last 5 years (2012–2017) and were isolated mainly from blood (*n* ≥ 361) and other deep‐seated infections, tissues and/or tips of invasive devices than from urine (*n* = 33) and ear discharge (*n* = 22) (Figure 3) (*p*‐value < .0001).

Patients infected or colonized with *C. auris* almost always presented with several other underlying health conditions or comorbidities including diabetes (*n* ≥ 52), sepsis or blood stream infections (BSI) (*n* ≥ 48), pulmonary diseases/pneumonia (*n* ≥ 39), chronic/acute kidney failure/pathologies, transplants etc. (*n* ≥ 32), immunosuppressive conditions (*n* ≥ 29), solid tumor/malignancies (*n* ≥ 26), cardiovascular diseases (*n* ≥ 24), chronic otitis media (*n* ≥ 18), liver disease (*n* ≥ 14) (Figure 2) etc. (*p*‐value < .0001). Many of the *C. auris* infections occurred in hospitalized patients on prior broad‐spectrum antibiotics and with invasive medical devices and/or procedures such as central venous catheter (CVC), arterial line, urinary catheter, parenteral nutrition, abdominal surgery, immunosuppressive agents etc. (Azar et al., 2017; Ben‐Ami et al., 2017).

Out of 316 patients, 94 were recorded as demised, which translated into 29.75% crude mortality rate (*p*‐value = .0488). Crude mortality per country showed that *C. auris* infections resulted in 33.33% to 100% crude mortality worldwide, with the least (33%) being recorded in South Africa and Israel; *p*‐value = .1789 (Figure 2c).

As shown in Table 2 and Figure 3, C*. auris* has been isolated from patients of both sexes and of all age groups. However, preterm or low‐birth weight infants as well as geriatrics are known to be highly at‐risk patients due to their weaker immune systems, such that they have high mortality risk upon being infected with *C. auris* (Chowdhary et al., 2013; Newnam & Harris‐Haman, 2017; Ruiz Gaitán et al., 2017; Schelenz et al., 2016; Schwartz & Hammond, 2017; Tsay et al., 2017). As geriatrics are more prone to be hospitalized in acute‐care hospitals or long‐term care facilities, it is more likely that they will be exposed to *C. auris* infections reported from healthcare centers.

Risk factors associated with *C. auris* infections are consistently the same in almost all the reported cases worldwide and these include the presence of catheters (urinary, central venous), arterial line, parenteral nutrition, invasive medical procedures (surgeries) and devices, mechanical ventilation, hospital and intensive care unit (ICU) stays, prior or continual exposure to broad spectrum antifungal or antibiotic therapy, or comorbid disease conditions such as diabetes mellitus and HIV/AIDS (Al‐Siyabi et al., 2017; Ben‐Ami et al., 2017; Calvo et al., 2016; Chowdhary et al., 2013, 2014; Lee et al., 2011; Lockhart et al., 2017; Mohsin et al., 2017; Morales‐Lopez et al., 2017; Rudramurthy et al., 2017; Ruiz Gaitán et al., 2017; Schelenz et al., 2016; Tsay et al., 2017; Vallabhaneni et al., 2016). It is obvious from these risk factors that invasive devices or procedures easily result in the introduction of and re‐infection with *C. auris* in most patients, and the removal of catheters resolved several candidemia (Chowdhary et al., 2014; Lee et al., 2011; Ruiz Gaitán et al., 2017). Hence, removal of catheters is a necessary first‐line strategy for managing and treating acute, recurring and persistent *C. auris* infections (Chowdhary et al., 2014; Lee et al., 2011; Ruiz Gaitán et al., 2017).

Moreover, the suppression of the immune system of patients with immunosuppressive agents such as steroids and malignancies or medical procedures that require such agents, specifically during organ transplants (Azar et al., 2017), also reduces the ability of the immune system to prevent the easy dissemination of *C. auris* invasive infections.

In addition, broad‐spectrum antimicrobials clear away nonpathogenic but important bacteria and fungi that offer competitive inhibition to *C. auris* pathogens, allowing the latter to proliferate freely. Thus, antimicrobial stewardship has been advised to prevent the proliferation of *C. auris* and related species (Ben‐Ami et al., 2017; Chakrabarti et al., 2015; European Centre for Disease Prevention and Control, 2016). Contact precautions are advised by the CDC (Centers for Disease Control and Prevention, 2017a, b; Tsay et al., 2017; Vallabhaneni et al., 2016) because close contact with an infected patient as well as being in the same hospital or ward is a risk factor for colonization or infection with *C. auris* (Schelenz et al., 2016; Tsay et al., 2017; Vallabhaneni et al., 2016).

*Candida auris* was first isolated from the ear in 2009 (Kim et al., 2009; Satoh et al., 2009), but it has subsequently been reported mostly in BSIs or sepsis and deep‐seated invasive infections (Figure 2). Hence, *C. auris* infections are currently associated with candidaemia, high mortalities (Figure 3c), persistent fungemia and therapeutic failure as they are difficult to clear from the blood even when they are susceptible (Ben‐Ami et al., 2017; Chowdhary et al., 2013, 2014; Vallabhaneni et al., 2016). In the first organ‐transplantation‐associated *C. auris* infection case, Azar et al. (2017) described the dangers involved in undertaking organ transplantation without prior investigation into the donor's clinical history and species of all *Candida* identified on the organ (Azar et al., 2017). In India and other areas, *C. auris* candidaemia ranges between 5% and 30% of all candidaemia cases (Calvo et al., 2016; Chowdhary et al., 2013, 2014; Rudramurthy et al., 2017) reported in selected hospitals. These show the rapid emergence of *C. auris* as a lethal pathogen and nosocomial threat. The true prevalence of *C. auris‐*mediated candidaemia could be higher if they are rightly detected.

In summary, *C. auris* has been isolated from both sexes in 16 countries and five continents worldwide, with risk factors ranging from the presence of invasive devices to immunocompromised conditions.

## 7 DIAGNOSTICS AND TYPING METHODS

Meta‐analysis showed that conventional PCR was the most used diagnostic tool in terms of number of studies (29/38) and collective sample size (*n* ≥ 484; 30.38%). Vitek 2 Yeast ID system was the second most common platform used per study (20/38) and third in terms of total sample size analyzed (*n* ≥ 190; 11.93%) while Bruker MALDI‐TOF MS was second in terms of total sample size analyzed (*n* ≥ 223; 14.00%) and third most used instrument in all the studies (10/38) (*p*‐value = .002). Detailed statistics on the diagnostic tools used in detecting and typing *C. auris* are comprehensively summarized in Tables 3, 4.

**Table 3:** Relative efficiencies of various diagnostics used for the identification of *Candida auris*

Diagnostic tool (usage frequency, *n* = 38)	Combined sample size	Sensitivity (%)	Specificity (%)	Turnaround time (hr)	Relative cost	Skill level required	References
Phenotypic and/or culture‐based media or methods							
CHROMagar *Candida* at 40–42°C (9/38)	37	40–73	0	24–48	Cheaper	Minor	Azar et al. (2017), Ben‐Ami et al. (2017), Chowdhary et al. (2014), Emara et al. (2015), Khillan et al. (2014), Kumar et al. (2015), Morales‐Lopez et al. (2017), Ruiz Gaitán et al. (2017), Satoh et al. (2009)
CHROM *Candida* supplemented with Pal's medium at 37–42 °C (1/38)	15	100	100	24–48	Cheaper	Minor	Kumar et al. (2017)
MAST ID CHROMagar *Candida* at 42°C (1/38)	1	0	100	24–48	Cheaper	Minor	Emara et al. (2015)
BBL Mycosel agar ± cycloheximide (2/38)	9	0	100	24–48	Cheaper	Minor	Emara et al. (2015), Ruiz Gaitán et al. (2017)
Brilliance *Candida* Agar (1/38)	50	0	0	24–48	Cheaper	Minor	Schelenz et al. (2016)
Salt Sabouraud broth with dextrose (1/38)	77	100	≤100	24–48	Cheapest	Minor	Welsh et al. (2017)
Salt Sabouraud broth with dulcitol/mannitol (1/38)	77	100	100	24–48	Cheapest	Minor	Welsh et al. (2017)
Salt Yeast Nitrogen base broth + dulcitol/mannitol (1/38)	77	100	100	24–48	Cheapest	Minor	Welsh et al. (2017)
Commercial identification instruments/kits							
API 20C (7/38)	48	0	0	18–72	Cheap	Minor	Chowdhary et al. (2013), Larkin et al. (2017), Lee et al. (2011), Magobo et al. (2014), Mohsin et al. (2017), Morales‐Lopez et al. (2017), Ruiz Gaitán et al. (2017)
AuxaColor^™^ 2 (1/38)	8	0	0	24–48	Cheap	Minor	Ruiz Gaitán et al. (2017)
Microscan Walkaway & AutoSCAN 4 (1/38)	17	0	0	(24+) 2–≤24	Expensive	High	Morales‐Lopez et al. (2017)
BD Phoenix Yeast ID panel (3/38)	24	0	0	(24+) 4–15	Expensive	High	Al‐Siyabi et al. (2017), Kim et al. (2016), Morales‐Lopez et al. (2017)
VITEK 2 YST ID (20/38)	190	0	0	(24+)	Expensive	High	Azar et al. (2017), Ben‐Ami et al. (2017), Calvo et al. (2016), Chakrabarti et al. (2015), Chatterjee et al. (2015), Chowdhary et al. (2013), Emara et al. (2015), Khillan et al. (2014), Kim et al. (2009, 2016), Larkin et al. (2017), Lee et al. (2011), Magobo et al. (2014), Morales‐Lopez et al. (2017), Oh et al. (2011), Rudramurthy et al. (2017), Sarma et al. (2013), Satoh et al. (2009), Sharma et al. (2015), Shin et al. (2012), Wattal et al. (2017)
VITEK MS MALDI‐TOF (4/38)	28	100	100	≤12	Very expensive	High	Azar et al. (2017), Kim et al. (2016), Ruiz Gaitán et al. (2017), Wattal et al. (2017)
Bruker Biotyper MALDI‐TOF (10/38)	223	100	100	≤12	Very expensive	High	Azar et al. (2017), Borman et al. (2016, 2017), Ghosh et al. (2015), Kathuria et al. (2015), Kim et al. (2016), Mohsin et al. (2017), Prakash et al. (2016), Schelenz et al. (2016), Schwartz and Hammond (2017)
Molecular‐based methods							
Conventional PCR (29/38)	484	100	100	2.5	Expensive	Very high	Ben‐Ami et al. (2017), Borman et al. (2016, 2017), Calvo et al. (2016), Chakrabarti et al. (2015), Chatterjee et al. (2015), Chowdhary et al. (2013, 2014), Emara et al. (2015), Ghosh et al. (2015), Kathuria et al. (2015), Khillan et al. (2014), Kim et al. (2009, 2016), Kordalewska et al. (2017), Kumar et al. (2015), Larkin et al. (2017), Lee et al. (2011), Magobo et al. (2014), Mohsin et al. (2017), Oh et al. (2011), Prakash et al. (2016), Rudramurthy et al. (2017), Ruiz Gaitán et al. (2017), Sarma et al. (2013), Satoh et al. (2009), Sharma et al. (2015), Shin et al. (2012), Wattal et al. (2017)
AFLP (4/38)	184	100	100	2.5–4	Expensive	Very high	Calvo et al. (2016), Chowdhary et al. (2013), Prakash et al. (2016), Schelenz et al. (2016)
Real‐time PCR (1/38)	140	100	100	2	Expensive	Very high	Kordalewska et al. (2017)
WGS (6/38)	160	100	100	8–72	Very expensive	Highest	Centers for Disease Control and Prevention (2017a), Chatterjee et al. (2015), Lockhart et al. (2017), Schwartz and Hammond (2017), Tsay et al. (2017), Vallabhaneni et al. (2016)

**Table 4:** Usage characteristics and analyzed sample sizes per diagnostic tool

Methods/tools	Descending order of usage frequency	Methods/tools	Descending order of combined sample size
Conventional PCR	29	Conventional PCR	484
Vitek 2 Yeast ID	20	Bruker Biotyper MALDI–TOF (10/38)	223
Bruker Biotyper	10	Vitek 2 Yeast ID	190
CHROMagar	9	AFLP	184
API 20C	7	WGS	160
WGS	6	Real‐time PCR	140
Vitek MS	4	Salt SAB/NBB	77
AFLP	4	Brilliance *Candida* Agar	50
		API 20C	48
		CHROMagar	37

The greatest hindrance to effective detection of *C. auris* in most microbiology laboratories is misidentification by available commercial identification platforms or systems such as the Vitek Yeast ID Panel, Microscan Walkaway, BD Phoenix, API 20C, Auxacolor, CHROMagar, etc. as *C. haemulonii, Candida famata, C. kefyr, C. duobushaemulonii, C. pseudohaemulonii, C. krusei, Rhodotorula glutinis* etc. (Table 1). Furthermore, without an updated database (Mizusawa et al., 2017; Wattal, Oberoi, Goel, Raveendran, & Khanna, 2017), it is impossible for the currently reliable and often used MALDI‐TOF MS systems, the Bruker Biotyper and the Vitek 2 MS, to correctly identify *C. auris* (Tables 3, 4) (Kordalewska et al., 2017). As well, discrepancies between MICs obtained from Vitek 2 and the CLSI MBD method have been reported for antifungal agents such as AMB, azoles, and echinocandins (Arendrup et al., 2017; Kathuria et al., 2015; Khillan et al., 2014). This is a serious observation as the Vitek 2 is a commonly used instrument for measuring the MICs of various antifungals against *C. auris* (Tables 3, 4). Although Shin et al. (2012) have argued that the Vitek 2 was better than the CLSI and EUCAST MBD protocols in detecting AMB resistance, particularly as the latter two methods yield very narrow AMB MICs that are unable to efficiently discriminate between AMB susceptible and resistant isolates, the Vitek 2 should not be used alone to report on the susceptibility of *C. auris* strains (Shin et al., 2012). This is particularly important as wrong susceptibility results can result in fatal consequences (Chowdhary et al., 2014; Kathuria et al., 2015; Kumar et al., 2015; Ruiz Gaitán et al., 2017; Vallabhaneni et al., 2016). For now the gold standard for *C. auris* MICs is the CLSI MBD protocol, which is the most widely used (Arendrup et al., 2017).

### 7.1 Diagnostic tools: culture‐based methods

Welsh et al. (2017) recently reported of two novel in‐house diagnostic broths they designed to efficiently screen for and detect *C. auris* from clinical and environmental specimens with relative ease, 100% specificity and sensitivity, and low cost. These broths, consisting of 10% salt, gentamicin, chloramphenicol and either dulcitol, mannitol or dextrose in Sabouraud broth or Yeast Nitrogen base (YNB), could inhibit the growth of all other species when cultivated at 42°C. However, when the Sabouraud broth with dextrose was used and cultured at a lower temperature, *C. glabrata* could also grow as it has high salinity tolerance. This easy‐to‐prepare and cheaper broth has been useful in controlling the spread of *C. auris* in the US and other countries (Welsh et al., 2017). Thus, its adoption in other laboratories will facilitate the easy and quicker detection of this problematic pathogen.

Kumar et al. (2017) combined two culture media, CHROMagar *Candida* media supplemented with Pal's medium, to perfectly distinguish between *C. auris* and *C. haemulonii*. Pal's medium was originally designed for the identification of *Cryptococcus neoformans* and has been useful in distinguishing *C. albicans* from *Candida dubliniensis*. On this merged medium, *C. auris* produced no pseudohyphae, grew at 42°C, and had confluent growth of white‐cream colored smooth colonies while *C. haemulonii* did not grow at 42°C, had pseudohyphae, and showed poor growth of smooth light‐pink colonies. This method, while very sensitive and specific (100%) if used for only these two species, is limited by the fact that an initial identification by available commercial systems to rule out other nonalbicans species of *Candida* is required (Kumar et al., 2017).

In an earlier work, Shin et al. (2012) used 38 species of *Candida* including 20 *C. auris* isolates to evaluate the capacity of five phenotypic tests namely, E‐test on Mueller‐Hinton agar supplemented with glucose and methylene blue (E‐test‐MH), E‐test on RPMI agar supplemented with 2% glucose (E‐test‐RPG), Vitek 2, as well as CLSI and EUCAST MBD protocols to determine AMB resistance in vitro. The E‐test‐MH method was adjudged the best in detecting AMB resistance followed by the Vitek 2 among *C. haemulonii* and *C. auris*. The CLSI and EUCAST MBD protocols yielded very narrow AMB MICs, which made them unable to efficiently discriminate between AMB susceptible and resistant strains (Shin et al., 2012). Further tests will be necessary to confirm this preliminary finding.

### 7.2 Diagnostic tools: MALDI‐TOF MS

The inefficiencies of available diagnostic tools in detecting or misidentifying *C. auris* are already mentioned above (Tables 3, 4). Using an updated research use only (RUO) library or database, which can be updated in‐house, the two available MALDI‐TOF MS platforms, the commonly used Bruker Biotyper^™^ and the lesser used Vitek MS, can detect *C. auris* with 100% sensitivity and specificity within a few minutes (Table 3). The Bruker Biotyper^™^ database 3.1 has spectra of three *C. auris* strains (Kathuria et al., 2015). Grenfell et al. (2016) showed that adding ClinProTools to the Flex Analysis provided higher discriminatory power in detecting biomarker peaks (Grenfell et al., 2016). Several researchers have also reported of the higher efficiency of the Bruker Biotyper over the Vitek 2 MS in detecting *C. auris* and other nonalbicans species of *Candida,* even with an updated database (Ghosh et al., 2015; Grenfell et al., 2016; Kim, Kweon, Kim, & Lee, 2016). The MALDI‐TOF MS has thus been used to reidentify 90 *C. auris* isolates out of 102 strains initially misidentified as *C. famata* and *C. haemulonii* by Vitek 2 (Kathuria et al., 2015). Prakash et al. (2016) and Girard et al. (2016) have both used the MALDI‐TOF MS to type *C. auris* isolates and found it to be as equally effective as genotypic tools such as amplified fragment length polymorphism (AFLP) and multilocus sequence typing (MLST), which are considered gold standards in molecular typing (Girard et al., 2016; Prakash et al., 2016). The MALDI‐TOF MS also holds the potential to discriminate between resistant and susceptible *C. auris* strains as done for CRE (Osei Sekyere, Govinden, & Essack, 2015), and should be further investigated to unleash this potential for MDR *C. auris* testing.

In terms of specimen preparation protocols, Ghosh et al. (2015) showed that the on‐plate formic acid extraction method is the most cost and time efficient (Ghosh et al., 2015). Mizusawa et al. (2017) also observed that the direct extraction method enabled the perfect detection of *C. auris* on the Vitek MS system while the full‐length or partial extraction method was necessary for 100% identification by the Bruker MS system (Mizusawa et al., 2017). Using the direct on‐plate extraction method resulted in only 50% identification of *C. auris* with low score match, with 50% being unidentified (Mizusawa et al., 2017). Girard et al. (2016) also used the direct smear protocol to identify *C. auris* with the Vitek MALDI‐TOF MS (Girard et al., 2016).

### 7.3 Diagnostic tools: PCR, real‐time PCR and whole genome sequencing (WGS)

The use of conventional PCR to amplify the ITS and/or D1/D2 DNA sequences, followed by sequencing of the amplicons is currently the gold‐standard and most commonly used technique to identify, confirm the identity and type *C. auris* strains (Tables 3, 4) with 100% specificity and sensitivity, and shorter turnaround time (Kordalewska et al., 2017). Recently, Kordalewska et al. (2017) developed a conventional PCR and real‐time assay that could respectively identify *C. auris* as well as *C. auris, C. duobushaemulonii* and *C. lusitaniae* with 100% sensitivity and specificity, and shorter turnaround time of 2.5 and 2 hr respectively. This protocol was also used in direct colony PCR to achieve the same optimum results. Either gel electrophoresis (for conventional PCR) or melting temperature (Tm) analysis (real‐time PCR) was used for final confirmation or differentiation of the results respectively. The amplicons covered a fragment of 5.8S, ITS2 and a part of 28S ribosomal DNA using CauF/R primers, which yielded a 163 bp long (conventional) PCR amplicon for *C. auris*. Further, CauRe1R primers (real‐time PCR) selectively amplified regions in either *C. auris, C. duobushaemulonii, C. haemulonii* or *C. lusitaniae*. The limit of detection of these assays were 10 CFU/reaction (Ct = 28.61 ± 0.25) for *C. auris‐*specific assays and 1,000 CFU/reaction (Ct = 27.83 ± 0.87) for *C. auris‐*related species (Kordalewska et al., 2017).

Besides using the sequenced amplicons to identify an isolate as *C. auris* by comparing the sequence to available sequences at GenBank, they can also be used in phylogenetic analysis to draw evolutionary or phylogenetic trees. These phylogenetic dendrograms has been instrumental in tracing the sources and clonality of the isolates in relation to other isolates from the same or different hospital, region, or country. Other PCR‐based typing tools such as AFLP and MLST have been used to identify and type *C. auris* strains (Tables 2, 3, 4). As well, other molecular but non‐PCR‐based or restriction enzyme‐based techniques such as PFGE and REAG‐N have been used occasionally to aid in the typing of *C. auris* (Oh et al., 2011). However, these above‐mentioned (PCR‐based and non‐PCR‐based) typing tools are labor intensive with longer turnaround times.

WGS is increasingly being used to aid in the simultaneous identification and typing or evolutionary analysis of *C. auris* cases (Chatterjee et al., 2015; Lockhart et al., 2017; Sharma et al., 2016; Tsay et al., 2017; Vallabhaneni et al., 2016). Due to its higher resolution, it can provide a better evolutionary and epidemiological analysis of *C. auris* cases than all other methods within a comparatively short turnaround time (8–72 hr), except that it is more expensive and requires higher skill and data processing capacity (Table 3, 4) (Lockhart et al., 2017).

### 7.4 CLSI and EUCAST MIC protocols

Arendrup et al. (2017) used 123 *C. auris* isolates of international origin to evaluate the MIC of common antifungals as obtained by the most commonly used CLSI MBD protocol and the less used EUCAST MBD protocol. They established a good correlation between both methods for FLZ and VRZ MICs. However, lower MICs were obtained by the EUCAST protocol for AMB, ANF, MCF, and PSZ. In terms of geometric MIC, there were slightly different values except for AMB for which the EUCAST MIC values were higher. Further experimentation with a larger number of isolates will be necessary to confirm this finding. Thus, although the MIC differences from both protocols are relatively minor, researchers should be mindful of the specifics when comparing MICs obtained from both methods (Arendrup et al., 2017). Notwithstanding these little differences in MIC values, it is expected that the CLSI protocol will continue to hold preeminence among researchers because of the large number of available MIC data generated from this protocol, which will facilitate easy comparison with already available data from other works.

The authors observed that the collective MIC values from any population will be influenced by the presence of wild‐type and nonwild‐type colonies as well as by the collective resistance mechanisms of the various strains (Arendrup et al., 2017). This might explain the variable resistance of *C. auris* to the other azoles besides FLZ. A low acquired resistance to AMB and echinocandins was recorded in the *C. auris* strains.

Summing it up, PCR and MALDI‐TOF are the commonly used diagnostic tools and the CLSI MBD remains the most commonly used protocol for MIC determination.

## 8 MOLECULAR EPIDEMIOLOGY

While the above‐mentioned methods have enabled the easy description of the molecular epidemiology and phylogenetic relationship between strains of the same or different hospitals and/or countries, their resolution power is relatively weaker than that of WGS, which has recently been used by Lockhart et al. (2017) to comprehensively describe the genomic evolution of 53 *C. auris* strains from India, Pakistan, South Africa and Venezuela. A further retrospective analysis of historical isolates (*n* = 15,271) from a SENTRY surveillance program showed that *C. auris* is less likely to have emerged prior to 2009 (Lockhart et al., 2017).

Due to misidentification of *C. auris* by most commercial identification systems and the nonspecies identification of many species of *Candida* in many mycology laboratories, the true prevalence and epidemiology of *C. auris* infections in most countries and the world is not known and is likely to be underestimated than overestimated (Kordalewska et al., 2017; Todd, 2017). Moreover, blood, fluid and tissue cultures for detecting *C. auris* grow slowly and they could be falsely negative in cases of low‐level or intermittent candidaemia (Todd, 2017). The molecular epidemiology of all reported *C. auris* cases are described below under their continents and countries according to the order of detection.

### 8.1 Far East Asia: Japan and South Korea

The earliest *C. auris* case was misidentified and undetected as far back as 1996 in South Korea (Lee et al., 2011), prior to the first reported case of *C. auris* by Satoh et al. (2009), which was isolated from a 70‐year old female Japanese patient. Satoh et al. (2009) were thus the first to describe and name the new pathogen as *C. auris* due to its closer phylogenetic, phenotypic and genotypic (Table 1) relationship to the *Candida* genus and its isolation from the ear. Using the D1/D2 and ITS sequences, they showed that this new pathogen phylogenetically clustered in the *Metschnikowiaceae* clade (Satoh et al., 2009). Thus far, this first work by Satoh et al. (2009) is the only reported case of *C. auris* in Japan to date. Later in the same year, Kim et al. (2009) also reported of a novel yeast species with close phenotypic similarity to *C. haemulonii* from the ear of 15 otitis media patients who visited five hospitals in South Korea between 2004 and 2006. These historical isolates, some of which were later found to be clonally related (Oh et al., 2011), were actually *C. auris,* with elevated FLZ, VRZ and AMB MICs or resistance.

Thus, it is obvious that *C. auris* first appeared in South Korea as early as 1996 (Table 2), but was misidentified and undescribed until Satoh et al. (2009) did so in Japan. Moreover, the Japanese isolate was later found to be very closely related phylogenetically to some of the isolates from South Korea (Ben‐Ami et al., 2017; Mohsin et al., 2017; Schelenz et al., 2016) and they all assimilated NAG while those from other countries did not (Table 1) (Prakash et al., 2016); thus, the possibility of transfer from South Korea to Japan or otherwise, should be investigated further. It is notable that almost all the isolates recovered from Japan and South Korea were from the ear (Table 2), except a few (*n* = 6) that were obtained from blood; at least two patients with candidaemia demised (Lee et al., 2011; Shin et al., 2012). Fortunately, no *C. auris* cases, either from the ear or blood (fungemia), have been reported in South Korea since 2013.

### 8.2 South Asia: India and Pakistan

Chowdhary et al. (2013) were the first to report on a clonal outbreak of *C. auris* candidaemia in India and worldwide involving 12 patients from two different hospitals in Delhi. Although reported in 2013, these isolates were collected between 2009 and 2011, and were clonally different from those from Japan and South Korea, suggesting an independent emergence of *C. auris* in India (Chowdhary et al., 2013). The isolates were highly resistant to FLZ and 50% of the patients died. Subsequently, India has recorded the largest number of *C. auris* candidaemia worldwide between 2009 and 2015 (Figure 2) (Table 2), including MDR isolates (Chakrabarti et al., 2015; Chowdhary et al., 2014; Prakash et al., 2016). There is a higher prevalence of *C. auris* infections in the public sector than private sector hospitals in India due to overcrowding and possible compromise in infection control (Rudramurthy et al., 2017), with *C. auris* prevalence ranging from 5% to 30% of all candidaemia cases in certain hospitals (Chowdhary et al., 2013; Rudramurthy et al., 2017). WGS, AFLP, MLST and MALDI‐TOF MS typing of several Indian strains showed their closer evolutionary or phylogenetic relationship and wider evolutionary or phylogenetic distance from those of other countries (Lockhart et al., 2017; Prakash et al., 2016). Lockhart et al. (2017) showed that the genomes of isolates from India differed from that of other countries by >10,000 SNPs, indicating the independent emergence of *C. auris* in this country (Lockhart et al., 2017). However, strains from the Pakistan, USA and UK have very close phylogenetic relationship with those from India, suggesting that they were possibly imported from India (Borman, Szekely, & Johnson, 2017; Vallabhaneni et al., 2016). Further, the first *C. auris* case in Canada was in a patient previously hospitalized in India (Schwartz & Hammond, 2017).

The higher prevalence of *C. auris* cases (Figure 2) and clonal outbreaks in India is very concerning, particularly as many were multidrug resistant and can spread to other countries as already reported (Borman et al., 2017; Schwartz & Hammond, 2017; Vallabhaneni et al., 2016). In one study, there was interhospital and intrahospital spread of clonal *C. auris* strains, even though there were no exchange of healthcare personnel between these hospitals and wards (Chowdhary et al., 2013). Resistance to FLZ has been found to be mediated by known mutations (Y132F and K143R) in *ERG11* (Lockhart et al., 2017). Recommended infection control protocols should be instituted and strictly followed to reduce the incidence of this MDR pathogen and its attendant mortalities (Centers for Disease Control and Prevention, 2017b; European Centre for Disease Prevention and Control, 2016; Schelenz et al., 2016; Todd, 2017).

The incidence of *C. auris* in Pakistan (*n* = 19) was first reported by Lockhart et al. (2017) and characterized using WGS (Table 2). The Pakistani isolates were collected between 2012 and 2015 and were found to be very closely related to those from India, with <60 SNPs between isolates. Notably, they resulted in very high crude mortalities (72%; 13/18) and also shared the same FLZ resistance mechanism (Y132F and K143R in *ERG11*) as that of the Indian strains (Lockhart et al., 2017). There are no reports of *C. auris* infections in Pakistan besides this, but the higher mortality rate is worrying. Further surveillance and prompt report of *C. auris* cases are necessary to detect cases as early as possible.

### 8.3 Middle East: Israel, Kuwait and Oman

Only a single report of *C. auris* candidaemia in six patients from two hospitals in Tel‐Aviv has been published to date in Israel (Ben‐Ami et al., 2017). These strains were collected between May 2014 and May 2015 and were phylogenetically different from those from East Asia, Africa, and the Middle East. They formed aggregates in the kidneys of mice infection models and were less virulent than *C. albicans,* but more virulent than *C. haemulonii*. The formation of aggregates by these Israeli strains is akin to that reported by Borman et al. (2016) and Sherry et al. (2017), and corroborates the assertion that some *C. auris* cells form aggregates, which are less virulent/pathogenic than nonaggregating ones and *C. albicans* (Ben‐Ami et al., 2017; Borman et al., 2016; Sherry et al., 2017). In addition, these strains were found to have higher ABC efflux activity than *C. glabrata* and *C. haemulonii,* which agrees with the enriched efflux genes reported by Chatterjee et al. (2015) in the *C. auris* genome and explains the MDR nature of this pathogenic yeast (Chatterjee et al., 2015). The phylogeny of these strains indicates that they emerged independently in Israel and were not imported as they did not have a close relationship with other isolates from the Middle East, South or East Asia (Ben‐Ami et al., 2017).

The first and only *C. auris* candidaemia case in Kuwait was reported by Emara et al. (2015). This case was in a 27‐year old woman with chronic renal failure who was admitted to the ICU in May 2014. The isolate was highly resistant to FLZ (MIC of >256 μg/ml), but was susceptible to AMB (MIC of 0.064 μg/ml), VRZ (MIC of 0.38 μg/ml), and CFG (MIC of 0.064 μg/ml). The patient involved unfortunately expired from multiorgan failure.

Two different research groups simultaneously reported of separate *C. auris* incidence at different hospitals in Oman in the same year (Al‐Siyabi et al., 2017; Mohsin et al., 2017), one of which described two clonal strains from two old (70 and 77 years) patients from the same hospital; one patient died (50% mortality) (Mohsin et al., 2017). Al‐Siyabi reported of five *C. auris* candidaemia cases involving mostly old patients in another hospital, of which three died (Al‐Siyabi et al., 2017). All the *C. auris* candidaemia cases were detected between August 2015 and February 2017, and the isolates expressed high resistance to FLZ; some patients died even though they were on ANF therapy. The onset of infection after hospitalization ranged from 22 to 62 days, showing that these candidaemia cases were nosocomially acquired. The phylogenetic relationship between the isolates from these two reports has not been undertaken, albeit this is necessary to show if the isolates from the two reports are clonally related, and if these cases were locally acquired or imported. The two clonally related isolates (Mohsin et al., 2017) however seem to have been locally acquired as the patients had never traveled outside Oman; notwithstanding, they clustered phylogenetically between isolates from India and UK. Further investigations might be necessary to show whether they had contacts with persons from some of these countries. However, no further reports of *C. auris* have been published from Oman.

### 8.4 Africa: South Africa

*Candida auris* candidaemia was detected in four male South African patients between October 2012 and October 2013, with high resistance to FLZ (Magobo, Corcoran, Seetharam, & Govender, 2014). Except for one patient aged 27, the ages of the patients were between 60 and 85 years. Lockhart et al. (2017) subsequently reported of an additional 10 isolates collected from South Africa between 2012 and 2014, which were closely related to each other with <70 SNPs, but very distant phylogenetically to those from Pakistan, India and Venezuela (Lockhart et al., 2017). Borman et al. (2017) showed that isolates from the UK had very close sequence similarity with those from South Africa, the first and only African country to report of a *C. auris* mediated candidaemia (Borman et al., 2017). Prakash et al. (2016) also reported that the South African strains clustered with other isolates of diverse geographical origin (Prakash et al., 2016).

### 8.5 Europe: Germany, Norway, Spain and UK

Reports of *C. auris* fungemia and colonization have been rare in continental Europe, with most *C. auris* cases being reported in the UK, which was the first country in Europe to report of *C. auris* incidence as well as a clonal outbreak involving 50 patients in a cardiothoracic center in London (Borman et al., 2016, 2017; Schelenz et al., 2016). The first reported case (candidaemia) of *C. auris* in the UK was in 2013 from three unrelated patients in distant geographical localities (Borman et al., 2016, 2017). PHE reports that at least 200 *C. auris* infection cases and colonizations have so far been recorded in the UK, although an estimated number of ≥103 cases were found in published literature (Figure 2) (Public Health England). The UK isolates were of two different phenotypes, which had distinct virulence characteristics: aggregate‐forming strains with lesser virulence and nonaggregate‐forming strains with higher virulence (Borman et al., 2016; Sherry et al., 2017). Borman et al. (2016) and Sherry et al. (2017) observed respectively that the UK isolates formed rudimentary and occasional pseudohyphae, a characteristic that has never been reported in any other *C. auris* strain worldwide (Table 1) (Borman et al., 2016; Sherry et al., 2017). Phylogenetic analysis showed that the UK strains were of international origin due to their close sequence similarity with strains from India, Japan, Kuwait, Malaysia, Korea, South Africa etc.

Further, Schelenz et al. (2016) found *C. auris* in the air, floors, beds, bedding materials, window sills, environmental surfaces as well as the nostrils, stools, axilla and groins of patients during the outbreak that occurred between April 2015‐July 2016 among 50 patients admitted to a cardiothoracic center in London, UK (Schelenz et al., 2016). Daily chlorhexidine washes could not eradicate *C. auris* colonization, possibly due to reinfection from patients’ bedding and clothing. Some patients on echinocandins still developed candidaemia, and the echinocandins could not clear/reduce *C. auris* colonization on the skin. A nurse caring for a heavily infected patient was also transiently colonized, but healthcare workers were generally not colonized. The persistence of *C. auris* on several surfaces and materials made their complete eradication from the hospital difficult despite thorough decolonization and decontamination with chlorhexidine‐based products and hydrogen‐peroxide vapor. Hence, positive patients can shed *C. auris* into the hospital environment, posing a risk of continuous transmission (Schelenz et al., 2016).

The first incidence of *C. auris* infections in continental Europe occurred in Spain among four patients, two of whom died (Ruiz Gaitán et al., 2017). ANF therapy could not clear candidaemia from one patient and all the strains were highly resistant to FLZ and resistant to VRZ. The ECDC (European Centre for Disease Prevention and Control, 2016) reported of single *C. auris* cases in Germany and Norway while Larkin et al. (2017) used two *C. auris* isolates obtained from the blood of a German male patient (Larkin et al., 2017). Such sporadic reports of *C. auris* infections should motivate public health officials to undertake periodic comprehensive surveillance of patients and hospital environments to determine the true prevalence of *C. auris* in Europe.

### 8.6 South America: Colombia and Venezuela

Three invasive *C. auris* reports, one from Colombia (*n* = 17) (Morales‐Lopez et al., 2017) and another two involving an outbreak case (*n* = 18) and additional cases (*n* = 5) from Venezuela (Calvo et al., 2016; Lockhart et al., 2017), have been published from South America. In the report from Colombia, most patients had a CVC (*n* = 16), a urinary catheter (*n* = 15) and a mechanical ventilator (*n* = 10), which are important risk factors for acquiring *C. auris* infections. A similar exposure to invasive instruments was found by Calvo et al. (2016) (Morales‐Lopez et al., 2017). The 17 isolates were from 17 patients from six different hospitals, and were collected from February through July 2017, with misidentification and delayed diagnosis resulting in the death of 35.2% of patients. The *C. auris* outbreak case in Venezuela resulted in *C. auris* being the 6th most common cause of fungemia in that hospital that year (Morales‐Lopez et al., 2017). Vallabhaneni et al. (2016) added that isolates from Illinois, USA, were closely related (identical sequence homology with <150 SNPs apart) to those from Venezuela (Calvo et al., 2016). On the contrary, Lockhart et al. (2017) showed that isolates from Venezuela emerged independently (Lockhart et al., 2017).

### 8.7 North America: USA and Canada

Beginning from 2013 when the first US case of *C. auris* was identified in New York, at least 232 *C. auris* candidaemia (*n* = 112) and colonization (*n* = 120) incidences have been recorded by the CDC (Figure 2) in nine states (Connecticut, Florida, Illinois, Indiana, Maryland, Massachusetts, New Jersey, New York, and Oklahoma) (Azar et al., 2017; Centers for Disease Control and Prevention, 2017a; Vallabhaneni et al., 2016), making it the largest recorded *C. auris* incidence so far, after India. Most of these reported candidaemia cases are from New York, which have been shown by WGS to be closely related to isolates from New Jersey and Maryland with <70 SNPs apart. Notably, there were overlapping stays at long‐term and acute care facilities within these states; for instance, isolates from Maryland and New Jersey differed by <10 SNPs (Tsay et al., 2017; Vallabhaneni et al., 2016). The lung donor‐derived *C. auris* isolate from Massachusetts was very closely related to that from Illinois, from where the lung donor was based (Azar et al., 2017; Tsay et al., 2017). WGS showed that isolates from the same state were very closely related to each other than to those from other states. As well, some cases from New York and all the cases in Oklahoma and Indiana were from patients who had been earlier treated abroad; some of the New York cases were in patients who had returned from the Middle East. It is thus believed that *C. auris* was introduced into the US from abroad followed by local transmission. For instance, isolates from Illinois were of the same clade as those from South America while those from New York and New Jersey were of the same clade as those from South Asia (Tsay et al., 2017).

Interestingly, only two *C. auris* cases had been reported by 2015 in the US, but this number shot up afterwards, suggesting a recent and rapid emergence or higher detection of this menace possibly due to increased awareness and education on detection methods. The minimum time from hospital admission to first isolation of *C. auris* was 18 days in the first seven cases; and five out of the seven patients died. In one case, a *C. auris* candidaemia that was susceptible to FLZ persisted in the patient even though the patient was on the same drug. In two cases, the *C. auris* candidaemia recurred for 3–4 months while some patients remained colonized months after first detection (Tsay et al., 2017). This shows that *C. auris* can easily spread through hospitals and patients from colonized or infected persons.

Only a single *C. auris* case has been detected in Canada in a patient who was initially admitted in a hospital in India (Schwartz & Hammond, 2017). The isolate was continually obtained from repeated swabbing of the same ear of the patient over a 6‐week period. No other *C. auris* isolate has been reported in Canada afterwards.

## 9 MANAGEMENT AND CLINICAL OUTCOMES (MORTALITIES)

An official management protocol for *C. auris* infections is yet to be concluded, an optimal antifungal agent(s) or dosing regimen for *C. auris* infections is not defined and CLSI/EUCAST breakpoints for this pathogen is wanting (Lepak et al., 2017), making researchers resort to that established for closely related species of *Candida* (Arendrup et al., 2017; Lockhart et al., 2017). Interestingly, the efficacy of this approach was recently established by Lepak et al. (2017) for FLZ and AMB using neutropenic disseminated candidiasis murine models infected with *C. auris* (Lepak et al., 2017). Pharmacokinetic and pharmacodynamic (PK/PD) data showed that the MICs breakpoints of these two antifungals for other species of *Candida,* will hold for *C. auris* as the concentrations (exposure target) associated with optimal outcomes were similar. Furthermore, there was a strong relationship between the PK/PD parameters and treatment outcome for each drug (including MCF) and the dose‐effect against *C. auris* for each drug was proportional to the MIC (Lepak et al., 2017). A tentative breakpoint for some selected antifungals has however been proposed by the CDC and was used in analyzing some of the data in this review (Table 2; Figure 2) (Centers for Disease Control and Prevention, 2017b; Schwartz & Hammond, 2017). Clinicians currently advise on the use of echinocandins empirical therapy when *C. auris* is expected as it is the current antifungal with the most effective activity against this pathogen. However, this can be changed when subsequent susceptibility results show other antifungals to be potent against the patient's isolate (Lepak et al., 2017; Todd, 2017).

Liposomal AMB has also been shown to be very effective against *C. auris* including the inhibition of biofilm formation and potency against *C. auris* biofilms. It is also used in combination therapies with an echinocandin (Azar et al., 2017; Emara et al., 2015; Ruiz Gaitán et al., 2017; Sherry et al., 2017; Vallabhaneni et al., 2016). The higher toxicity of AMB in comparison with the much tolerated azoles and echinocandins limits its use clinically (European Centre for Disease Prevention and Control, 2016). Due to the relatively higher efficacy of the other less used azoles such as ITZ, ISA, and PSZ as well as FCN against *C. auris,* they can still be used either alone or in combinations when susceptibility testing proves their potency. The emergence of novel antifungal drugs, SCY‐078 and VT‐1598, which have so far demonstrated 100% efficiency against *C. auris* infections is a welcoming news for clinicians as it bolsters the available antifungal arsenals (Anonymous, 2017; Berkow et al., 2017; CDC, 2017; Larkin et al., 2017). Particularly, SCY‐078 is not affected by common mutations in protein targets, is orally bioavailable and active against echinocandin‐resistant strains (Berkow et al., 2017). It is not advisable however, to offer antifungal therapy to colonized patients (Todd, 2017).

## 10 INFECTION CONTROL AND PREVENTION

The persistence and recurrence of *C. auris* in the hospital environment in the face of rigorous decontamination, disinfection and decolonization protocols, as occurred in the UK over a one‐year period, should be a wake‐up call to all infection control personnel in all healthcare centers (Schelenz et al., 2016). Due to issues with misidentification, it is necessary that all microbiology (mycology) laboratories update their commercial identification softwares to enable them to easily and efficiently identify *C. auris* cases. Specifically, *C. famata, C. haeumolonii, C. sake, C. krusei, R. glutinis*, etc. strains should be further analyzed with PCR or MALDI‐TOF to confirm they are not misidentified. Or where such systems are unavailable, to quickly transport such specimens to local or foreign reference laboratories, isolate the patient under contact precautions and start empirical echinocandin therapy while awaiting the outcome of the laboratory tests (Azar et al., 2017; Centers for Disease Control and Prevention, 2017a, b; Lepak et al., 2017; Todd, 2017). In cases of organ transplants, donor organs should be scrutinized for sterility from *C. auris* prior to transplantation (Azar et al., 2017). Antifungal stewardship is necessary and prophylactic antifungal therapy or broad‐spectrum antibiotics prescription should be administered with caution (Ben‐Ami et al., 2017; European Centre for Disease Prevention and Control, 2016) as they can select for resistant *C. auris* and other drug‐resistant *Candida* spp (Ben‐Ami et al., 2017; Chakrabarti et al., 2015).

Patients with *C. auris* infections, persons colonized with or suspected to have such infections or patients transferred from hospitals with a history of *C. auris* infections or outbreaks should be kept in separate wards under strict contact precautions as detailed by the CDC, ECDC, and PHE (Bishop et al., 2017; Centers for Disease Control and Prevention, 2017b; European Centre for Disease Prevention and Control, 2016; Public Health England, 2016; Schwartz & Hammond, 2017; Seiffert et al., 2014). Such contact precautions have proved effective in the containment of outbreaks by other multidrug‐resistant organisms, particularly CRE, and was useful in the UK *C. auris* outbreak case (Schelenz et al., 2016). Patients or healthcare workers coming in close contact with infected persons should also be placed under strict contact precautions until they consistently provide negative cultures over 3 weeks (Schelenz et al., 2016). The wards of patients found to be colonized or infected with *C. auris* should be thoroughly disinfected as described (Schelenz et al., 2016).

MICs should ideally be measured using the CLSI MBD protocol to ensure accurate susceptibility results, which can inform correct therapeutic choices (Khillan et al., 2014; Lepak et al., 2017). Clinicians should also consider removing CVCs and other catheters where possible as certain studies have found such options useful in resolving persistent candidaemia (Calvo et al., 2016; Lee et al., 2011; Ruiz Gaitán et al., 2017).

Besides culture‐based methods in surveillance studies, esterase activity as measured by a solid‐phase cytometer should be considered to enhance the detection of viable but nonculturable strains (Welsh et al., 2017). Hospital wards, bedding materials, beds, invasive and noninvasive medical devices, clothing of patients, skin and surface wounds etc. should be decontaminated, using chlorine‐based detergents such as chlorhexidine (0.2%–4%) and hydrogen peroxide vapor (Schelenz et al., 2016; Sherry et al., 2017). As well, chlorhexidine‐impregnated protective discs for all CVC exit sites can aid reduce line‐associated *C. auris* BSIs (Schelenz et al., 2016). Oral nystatin plus nasal ointments have proved effective in decolonizing healthy nasal carriers (Schelenz et al., 2016). Soap and handwashing followed by alcohol‐based hand sanitizer is recommended by PHE. Admission screening of patients from infected sites or areas, active surveillance to identify carriers and prompt notification of the clinical infection control team are important (European Centre for Disease Prevention and Control, 2016).

## 11 CONCLUSIONS, FUTURE PERSPECTIVES AND STUDY LIMITATIONS

It is evident from this review that *C. auris* infections are more commonly reported in India, the USA and the UK, with fewer or isolated cases in South America, Africa, and continental Europe. Phylogenetic data show the independent emergence of *C. auris* in several countries. Misidentification, intrahospital transmission, poor treatment outcomes and higher crude mortalities between 33.33% and 100% are associated with *C. auris* infections worldwide. An official treatment guideline for *C. auris* infections is lacking and empirical treatment involving an echinocandin is advised. Contact precautions and effective disinfection with chlorine‐based agents are advised for hospitals with *C. auris* cases. PCR and MALDI‐TOF MS remain the most efficient and commonly used diagnostic tools.

Todd (2017) has suggested that the variations in *C. auris*‐associated mortalities could emulate those of other emerging infections in which initial cases are most severe, but tend to drop in severity over time (Todd, 2017). It would be welcoming should this be the case with *C. auris*. Efficient identification tools such as the MALDI‐TOF MS, PCR and WGS are still beyond the reach of many mycology laboratories worldwide, defeating efficient and prompt detection, earlier initiation of therapy and effective surveillance of *C. auris* in hospitals. Without efficient detection, the true prevalence of this menace will never be known, effective management of potential cases will be elusive and mortalities will continually rise. Thus, designing a simple, low‐cost detection technique, kit or tool with a shorter turnaround time is the key to defeating this deadly pathogen. Furthermore, the possibility of this yeast also spreading into the community, farms and the general environment should not be lost sight of. Evidence from CRE and MCR‐1‐positive bacteria should advise mycologists of the potential of this yeast to also infect livestock.

This review was limited by the fact that several studies failed to detail the year, sex, age(s), mortality, antifungal MICs, total number of isolates and patients, comorbidities, and specimen of the reported *C. auris* infections. This made the meta‐analysis challenging as such studies had to be excluded.

## AVAILABILITY OF MATERIALS AND DATA

Supplementary data is included in this manuscript.

## AUTHOR CONTRIBUTIONS

JOS designed and undertook the meta‐analysis, systematically reviewed the literature and wrote the paper.

## ETHICAL APPROVAL

Not applicable.

## TRANSPARENCY DECLARATION

The author declares no conflict of interest in the publication of this manuscript.
