# *Candida auris*: A systematic review and meta‐analysis of current updates on an emerging multidrug‐resistant pathogen

**DOI:** 10.1002/mbo3.901

**Published:** 2019-08-13

**Authors:** John Osei Sekyere

Subsequent to the publication of the paper by Osei Sekyere (2018), an error has been identified with the calculation of the drug resistance rates of the various antifungal drugs. The resistance rates of *Candida auris* to fluconazole and other antifungals were calculated by dividing the resistant isolates by all the retrieved isolates (*n* = 742) and multiplying the ratio by 100 instead of dividing the resistant isolates by the total number of isolates for which antifungal susceptibility data were available. A recalculation has been done for all these antifungal agents. With this new alteration, the resistance rate of fluconazole is within the range reported worldwide for *C. auris*. The correct Figure 3c is given below:

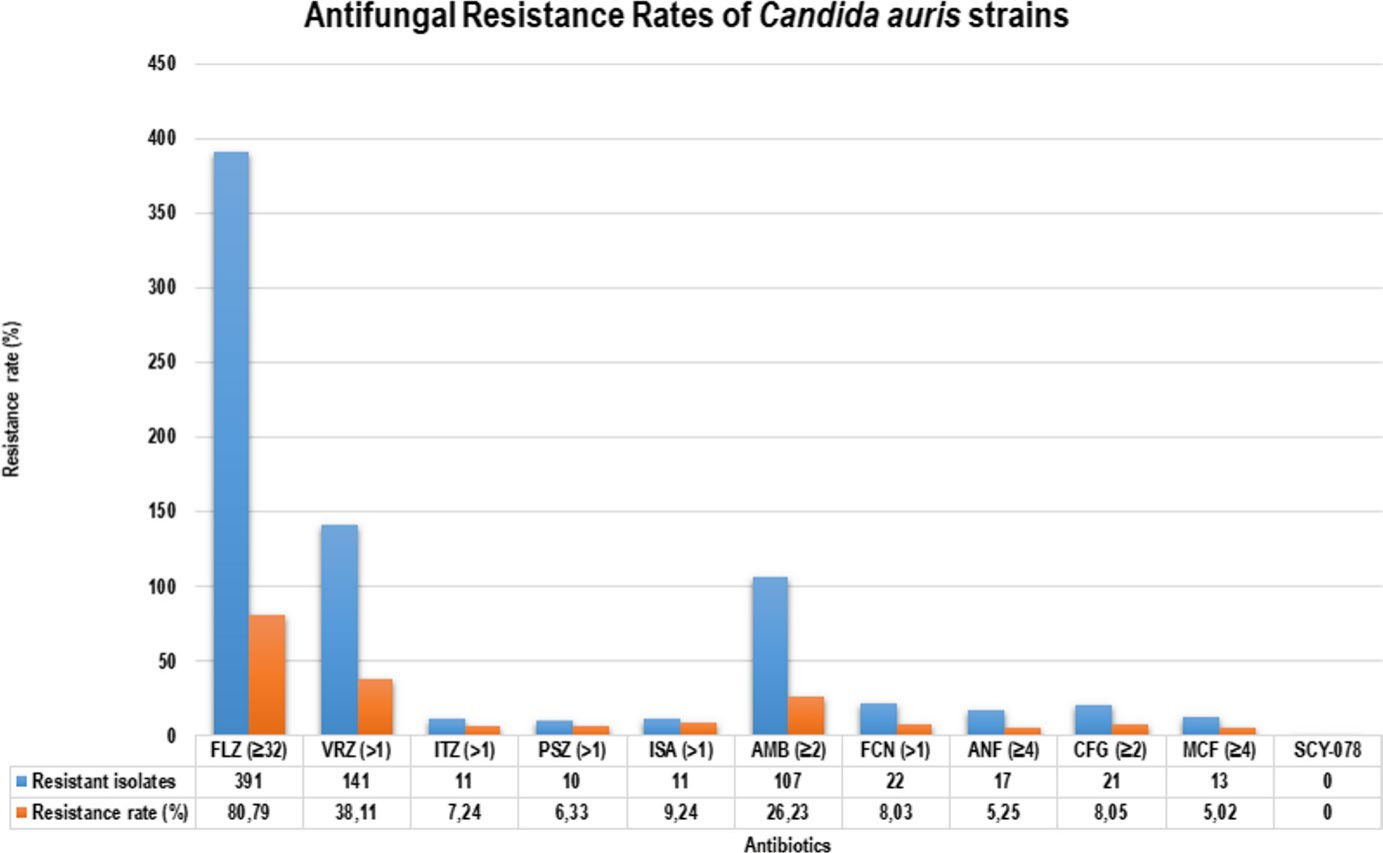

FLZ = fluconazole; VRZ = voriconazole; ITZ = itraconazole; PSZ = posaconazole; ISA = isoconazole; AMB = amphotericin B; FCN = flucytosine; ANF = anidulafungin; CFG = caspofungin; MCF = micafungin.

This error is sincerely regretted.
